# Robust automated prediction of the revised Vienna Classification in colonoscopy using deep learning: development and initial external validation

**DOI:** 10.1007/s00535-022-01908-1

**Published:** 2022-08-16

**Authors:** Masayoshi Yamada, Ryosaku Shino, Hiroko Kondo, Shigemi Yamada, Hiroyuki Takamaru, Taku Sakamoto, Pradeep Bhandari, Hitoshi Imaoka, Aya Kuchiba, Taro Shibata, Yutaka Saito, Ryuji Hamamoto

**Affiliations:** 1 Endoscopy Division, National Cancer Center Hospital, 5-1-1 Tsukiji, Chuo-ku, Tokyo, Japan; 2 Division of Medical AI Research and Development, National Cancer Center Research Institute, Tokyo, Japan; 3 Biometrics Research Laboratories, NEC Corporation, Kawasaki, Kanagawa Japan; 4 RIKEN Center for Advanced Intelligence Project, Cancer Translational Research Team, Tokyo, Japan; 5 Department of Gastroenterology, Portsmouth Hospitals University NHS Trust, Portsmouth, UK; 6 Biostatistics Division, National Cancer Center, Tokyo, Japan

**Keywords:** Deep learning, Artificial intelligence, Colonoscopy, Multi-class classification, External validation

## Abstract

**Background:**

Improved optical diagnostic technology is needed that can be used by also outside expert centers. Hence, we developed an artificial intelligence (AI) system that automatically and robustly predicts the pathological diagnosis based on the revised Vienna Classification using standard colonoscopy images.

**Methods:**

We prepared deep learning algorithms and colonoscopy images containing pathologically proven lesions (56,872 images, 6775 lesions). Four classifications were adopted: revised Vienna Classification category 1, 3, and 4/5 and normal images. The best algorithm—ResNet152—in the independent internal validation (14,048 images, 1718 lesions) was used for external validation (255 images, 128 lesions) based on neoplastic and non-neoplastic classification. Diagnostic performance of endoscopists was compared using a computer-assisted interpreting test.

**Results:**

In the internal validation, the sensitivity, specificity, positive predictive value (PPV), negative predictive value (NPV), and accuracy for adenoma (category 3) of 84.6% (95% CI 83.5–85.6%), 99.7% (99.5–99.8%), 90.8% (89.9–91.7%), 89.2% (88.5–99.0%), and 89.8% (89.3–90.4%), respectively. In the external validation, ResNet152’s sensitivity, specificity, PPV, NPV, and accuracy for neoplastic lesions were 88.3% (82.6–94.1%), 90.3% (83.0–97.7%), 94.6% (90.5–98.8%), 80.0% (70.6–89.4%), and 89.0% (84.5–93.6%), respectively. This diagnostic performance was superior to that of expert endoscopists. Area under the receiver-operating characteristic curve was 0.903 (0.860–0.946).

**Conclusions:**

The developed AI system can help non-expert endoscopists make differential diagnoses of colorectal neoplasia on par with expert endoscopists during colonoscopy.

(229/250 words).

**Supplementary Information:**

The online version contains supplementary material available at 10.1007/s00535-022-01908-1.

## Introduction

Artificial intelligence (AI) is increasingly being applied to medical images [1–3] and AI based on deep learning can achieve high diagnostic accuracy comparable to that of human experts [4–6]. We previously developed a system that detects early signs of colorectal cancer by applying deep learning to colonoscopy images [7].

During a colonoscopy, polypectomy is performed to reduce the incidence and mortality of colorectal cancer (CRC) [8–10]. Accurate endoscopic determination of colorectal polyp histology is essential for preventing unnecessary polypectomies and biopsy before endoscopic resection, setting appropriate surveillance intervals, and reducing the costs associated with the histopathologic assessment of polyps. Previous studies have found that expert endoscopists achieve high performance in optical diagnosis with ~ 90% sensitivity and 80–90% specificity, whereas community-based gastroenterologists achieve only ~ 70% sensitivity and 75% specificity [11, 12]. Furthermore, several prospective studies have noted lower sensitivity (70%) and specificity (80%) in differentiating malignant from benign colon polyps under white-light imaging (WLI) [13, 14].

To overcome these gaps and prevent missed diagnosis, we sought to develop a robust automated endoscopic differential diagnosis prediction system using deep learning. Deep learning models have been successfully applied in various computer vision tasks [15, 16]. In recent years, deep learning has also been applied in the field of endoscopy, including polyp detection in colonoscopy, differential diagnosis in upper gastrointestinal endoscopy, and determination of *Helicobacter pylori* infection status. Several prospective randomized control trials have been reported for the detection of colorectal tumors in colonoscopy with AI, and some programs have been approved as medical devices due to their usefulness in combination with AI [17]. Despite this progress, however, AI technology is still far from the point where correct diagnosis with AI eliminates the need for pathological evaluation.

To our knowledge, nearly all of these previous studies have used only two-category output, namely, neoplastic lesion versus non-neoplastic lesion or cancer versus erosion. Only one study reported a deep learning model for differential diagnosis in colonoscopy, but this model was designed to distinguish only two categories—adenomatous versus hyperplastic diminutive polyps—and lacked external validation data [18]. Given that colorectal carcinogenesis is a multistep process involving alterations of several oncogenes and tumor suppressor genes, a multi-class classification model for pathological categorization can be considered suitable for performing differential diagnosis in colonoscopy. In the present study, we developed and externally validated deep learning models that automatically predict a differential diagnosis according to the revised Vienna Classification [19] by using colonoscopy images of various pathological non-invasive lesions and adenocarcinomas of the colorectum. We aimed to develop and validate an AI system for multi-class classification along with a multi-step model of CRC carcinogenesis and compared its performance to that of endoscopists.

## Materials and methods

### Patients and colonoscopy image samples

This study was a multicenter, retrospective observational study using stored colonoscopy images. Inclusion criteria were patients who had colorectal neoplasms. Exclusion criteria were patients with advanced colorectal cancer (type 1–5 in Parris’ classification), inflammatory bowel disease, familial adenomatous polyposis, and patients who underwent chemotherapy and radiation therapy for colorectal cancer.

**Fig. 1:**
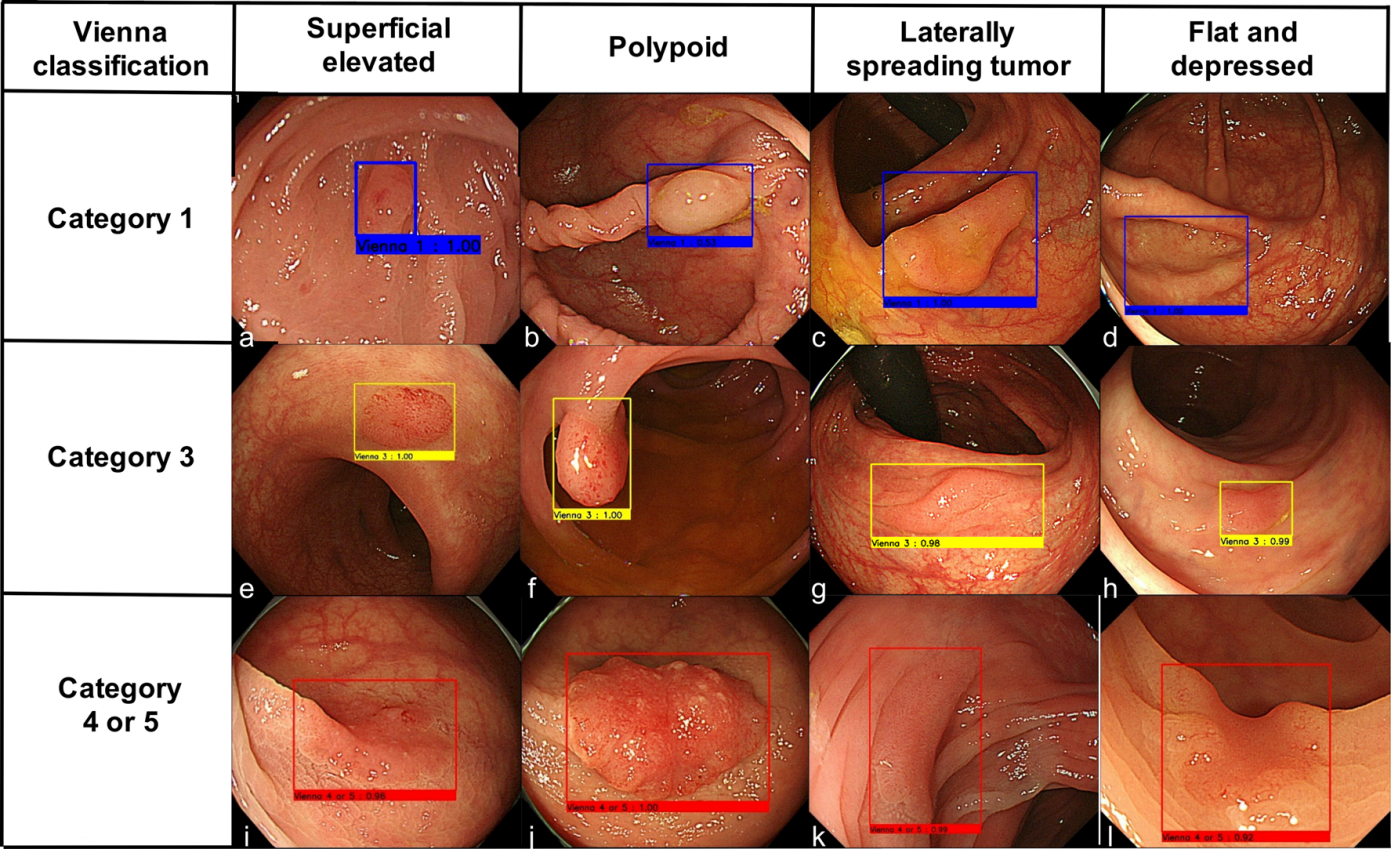
Representative images in white light imaging. **a**, **e**, **i** Superficial elevated type. **b**, **f**, **j** Polypoid type. **c**, **g**, **k** Laterally spreading tumor type. **d**, **h**, **i** Flat and depressed type. **a**, **b**, **c**, **d** Category 1 in the revised Vienna Classification. **e**, **f**, **g**, **h** Category 3 in the revised Vienna Classification. **i**, **j**, **k**, **l** Category 4/5 in the revised Vienna Classification

The images were captured using the following three modalities: WLI; equipment-based image-enhanced endoscopy (IEE), including narrow-band imaging (NBI) and blue laser imaging (BLI); and chromoendoscopy, which includes indigo carmine dye spraying and crystal violet staining (Fig. 1, Supplementary Figs. 1 and 2). All images were obtained using commonly used endoscopes (PCF-Q240ZI, CF-H260AZI, PCF-Q260AZI, CF-HQ290AI, and PCF-H290AZI, Olympus Optical Co., Tokyo, Japan; EC-580RD/M, EC-590MP, EC-590ZP, EC-590WM3, EC-600ZW/M, EC-600WM, and EC-L600ZP, Fujifilm Medical Co., Tokyo, Japan) and a standard video processor system (EVIS LUCERA, Olympus Optical; Advancia HD or LASEREO; Fujifilm Medical).

The characteristics of the lesions and the numbers of images used in the present study are summarized in Table 1 and Supplementary Table 1, respectively. All lesions in the training and validation sets were pathologically proven early-stage CRCs (Tis or T1) or precancerous lesions. The selected images had good image quality (less halation, no stool, etc.), were in focus, free of hemorrhage, showed a single lesion in each image, showed no devices, and were collected by the authors (MY, SR, HK, SY). All lesions in the images were manually annotated as regions of interest (ROIs) at their edges by two authors (HK, SY) and they were confirmed by an experienced endoscopist (MY).

**Table 1:** Number of images used in the deep learning training and validations

Revised Vienna Classification	Training	Hyperparameter tuning	Internal validation	External validation
Category 1				
Hyperplastic polyp or sessile serrated lesion, images (lesions)	7004 (1086)	1869 (274)	963 (198)	83 (68^a^)
WLI	1917 (868)	522 (219)	221 (127)	46 (34)
IEE (BLI/NBI)	2915 (945)	726 (237)	466 (168)	37 (34)
Chromoendoscopy*	2172 (557)	621 (150)	276 (106)	0 (0)
Category 3				
Low-grade adenoma/dysplasia images (lesions)	15693 (3489)	2652 (585)	5639 (1316)	172 (131^†^)
WLI	4567 (2741)	713 (453)	1586 (971)	98 (60)
IEE (BLI/NBI)	7036 (3137)	1242 (556)	2667 (1154)	74 (71)
Chromoendoscopy*	4090 (1556)	697 (271)	1386 (528)	0 (0)
Category 4/5				
High-grade adenoma/dysplasia or Submucosal invasive cancer images (lesions)	12629 (1075)	3164 (266)	1955 (204)	
WLI	3137 (942)	693 (216)	490 (162)	
IEE (BLI/NBI)	4492 (937)	1169 (235)	762 (176)	
Chromoendoscopy*	5000 (657)	1302 (155)	703 (112)	
Normal, images	11056	2805	5491	0
WLI	3650	898	2075	
IEE (BLI/NBI)	4594	1221	2061	
Chromoendoscopy*	2812	686	1355	
Total number of images	46382 (5650)	10490 (1125)	14048 (1718)	255 (128)

*WLI* white light imaging, *IEE* image-enhanced endoscopy, *BLI* blue laser imaging, *NBI* narrow-band imaging

*Chromoendoscopy includes indigo carmine dye spraying and crystal violet staining

^a^The 71 lesions were captured using both WLI and IEE images and were counted in both groups

### Building deep learning algorithms and internal validation

Among the collected still images of colonoscopies performed between January 2013 and December 2018, the following types of images were used to train the deep learning model in this study: revised Vienna classification, category 1, hyperplastic polyps (HP) or sessile serrated lesions (SSL); category 3, low-grade adenoma/dysplasia; category 4, high-grade adenoma/dysplasia; category 5.1, intramucosal carcinoma; category 5.2, submucosal invasive carcinoma; and normal images (NA) cropped from the non-diseased area of the lesion images [19]. All images were pathologically verified according to the revised Vienna Classification. In the case of heterogeneous histology, the higher category was preferentially adopted. The criterion for standard pathology was agreement on the histopathological diagnosis among three pathologists at our hospital (the training set and internal validation set). Based on the standard clinical treatment strategy (normal mucosa, no treatment; non-neoplastic lesions, case by case; benign neoplastic lesions, cold polypectomy or EMR, malignant lesions: EMR, ESD or surgery), categories in the AI system were set for categories 1, 3, and 4/5 of the revised Vienna Classification and NAs. The diagnosis of SSL was not mentioned in the revised Vienna Classification, but was diagnosed by a pathologist according to the World Health Organization classification and included in Category 1 of the Vienna Classification in the present study.

The collected dataset contains 51,550 images of 8,493 consecutive lesions, and 19,352 NAs were assigned to training, hyperparameter tuning, and internal validation sets in a ratio of about 5:1:1 (Table 1 and Supplementary Table 1). The training and hyperparameter tuning datasets were collected from 2013 to 2017, and the internal validation set was collected in 2018 at our division. The hyperparameter tuning set was used for setting adequate values of hyperparameters including the learning rate, batch size, number of iterations, momentum, and weight decay.

Deep learning algorithm—ResNet152 (Supplementary Fig. 3 and Supplementary Table 2)—were trained to learn the colonoscopy features of the disease [20]. Data augmentation (DA) was used to eliminate the class imbalance between the four categories (Supplementary Fig. 4) [21]. Detailed information is provided in the online supplemental materials. The category with the highest output score in the multi-class classification was adopted as the AI’s inference result. The diagnostic performance of the trained model for categories 1, 3, and 4/5 of the revised Vienna Classification and NAs and the inference speed were calculated using the internal validation set.

### External validation

An external validation study focusing on differentiation between neoplastic and non-neoplastic lesions was performed as in previous studies [18, 22]. All images collected between July 2020 and October 2020 from seven community hospitals in four prefectures of Japan excluding our hospital were assigned. Inclusion criteria were as follows: (1) patients elder than 20 y/o who underwent colonoscopy for fecal immunochemical testing positive, and surveillance after polypectomy, (2) patients who have macroscopic type 0 in Pari’s classification lesion. Colonoscopy was aimed at endoscopic resection, and patients who have inflammatory bowel disease or previous colonic surgery were excluded. Lesions pathologically proved hyperplastic polyp, sessile serrated lesion, adenoma, or adenocarcinoma were used for the external validation. Among them, images that had good image quality (less halation, no stool, etc.), were in focus, free of hemorrhage, showed a single lesion in each image, and showed no device were selected.

The external validation set was comprised 255 images of 128 lesions, including 83 images of non-neoplastic lesions (56 HP and 27 SSL images) and 172 images of neoplastic lesions (Table 1 and Supplementary Table 1). The diagnostic performance of the trained model for differentiation of neoplastic and non-neoplastic lesions and the inference speed were calculated on the graphics processing unit (NVIDIA GeForce RTX 2070) of a personal computer. The pathological information set was taken from the medical records of each participating institution.

To compare diagnostic yields between the AI system and the endoscopists, an observational study involving a computer monitor test was conducted, using all 255 images from the external validation set. The participating endoscopists were all employees of our hospital and were classified into the following groups: expert (≥ 5000 colonoscopies or certification by the Japan Gastroenterological Endoscopy Society; 4 endoscopists), fellow (< 5000 colonoscopies and no board certification; 3 endoscopists), and novice (< 1000 colonoscopies and no board certification; 5 endoscopists). The observers were blinded to both the histopathological diagnosis and clinical information, and the images were evaluated randomly to calculate the human diagnostic yield for each observer.

### T-distributed stochastic neighbor embedding (t-SNE) analysis

We analyzed the internal features of the fully trained ResNet152 model by using t-SNE analysis. Detailed information is provided in the online supplemental materials.

### Statistical analysis

The diagnostic performance of the trained model was evaluated by estimating the sensitivity, specificity, NPV, and PPV with their Clopper–Pearson exact 95% CIs. In the internal validation, accuracy, PPV, and NPV were calculated under the assumption that the ratio of lesions (including category 1, 3, and 4/5) to non-lesions (including NAs) was 60:40. The diagnostic performance was calculated on an image basis that had a confidence score of 0.9 or higher. Detailed information is provided in the online supplemental materials.

### Patient and public involvement

This study included no patient and public involvement.

## Results

### Representative depiction of the AI

Representative images of AI responses according to category and lesion morphology are shown in Fig. 1 and Supplementary Figs. 1 and 2 for the modalities WLI, IEE, and chromoendoscopy, respectively. In addition, a representative depiction of AI is shown in Supplementary Video 1.

### Characteristics of lesions

The clinicopathological characteristics of the lesions in the validation sets are shown in Supplementary Table 1. The numbers of images used for internal and external validation for each modality (WLI, IEE, and chromoendoscopy) are summarized in Table 1. The number of images stratified by tumor location, tumor size, and morphology are summarized in Supplementary Table 3. Supplementary Table 4 summarizes the details of the manufacturer and modality (WLI or IEE) used in the external verification set. Detailed information is provided in the online supplemental materials.

### Deep learning algorithm

On the basis of these results, we chose ResNet152 as the prediction model because it had the highest accuracy with the fastest inference time. Detailed information is provided in the online supplemental materials.

### Diagnostic performance of the AI system in the internal validation test

The diagnostic performance of the fully trained ResNet152 model in the internal validation test is summarized in Table 2. The model achieved an overall accuracy of 89.8% (95% CI 89.3–90.4%). The sensitivities for categories 1, 3, and 4/5 were 82.4% (95% CI 79.6–85.1%), 84.6 (95% CI 83.5–85.6%), and 79.8 (95% CI 77.7–81.8%), respectively, and the specificity was 99.7% (95% CI 99.5–99.8%). The positive predictive values (PPVs) for categories 1, 3, and 4/5 were 72.8% (95% CI 69.8–75.8%), 90.8% (95% CI 89.9–91.7%), and 70.4% (95% CI 68.2–72.5%), respectively, when calculated with an assumption that the ratio of lesions (including categories 1, 3, 4 and 5) to non-lesions (including NAs) was 60:40 based on a previous study [23]. The negative predictive values (NPVs) for categories 1, 3, and 4/5 achieve 91.2% (95% CI 90.7–91.8%), 89.2% (95% CI 88.5–90.0%), and 93.3% (95% CI 92.8–93.8%), respectively, when calculated with an assumption that the ratio of lesions to non-lesions was 60:40.

**Table 2:** Diagnostic performance of the AI system for predicting the pathology of early-stage colorectal cancers and precursor lesions in the internal validation

Revised Vienna Classification	Sensitivity %, (95% CIs)	Specificity %, (95% CIs)	Positive predictive value %, (95% CIs)	Negative predictive value %, (95% CIs)	Accuracy %, (95% CIs)
Category 1^*^	82.4	99.7 (99.5–99.8)	72.8	91.2	89.8 (89.3-90.4)
	(79.6–85.1)		(69.8-75.8)	(90.7-91.8)	
Category 3^a^	84.6		90.8	89.2	
	(83.5–85.6)		(89.9-91.7)	(88.5-90.0)	
Category 4/5^b^	79.8		70.4	93.3	
	(77.7–81.8)		(68.2-72.5)	(92.8-93.8)	
Overall	83.3		83.4	99.4	
	(82.4–84.1)		(82.5-84.3)	(99.2-99.7)	

*CIs* confidence intervals

*Vienna Classification Category 1 includes hyperplastic polyp or sessile serrated lesion

^a^Category 3 includes low-grade adenoma/dysplasia

^b^Category 4/5 includes high-grade adenoma/dysplasia or submucosal invasive cancer

Results of subgroup analysis of sensitivity (by tumor location, tumor size, and morphology), raw data for the diagnostic performance of the AI system, and diagnostic performance stratified by modality and manufacturer are summarized in Supplementary Table 5, Supplementary Table 6, and Supplementary Table 7, respectively. Detailed information is provided in the online supplemental materials.

Among all 14,048 images, 13.2% (1855 images) were diagnosed with a confidence score lower than 0.9 and excluded from the analysis. The distribution was differed between lesion or NA: 22% in category 1, 20% in category 3, 23% in category 4 or 5, and 1% in NA.

### External validation of AI performance in differentiating neoplastic and non-neoplastic lesions and comparison with endoscopists

**Fig. 2:**
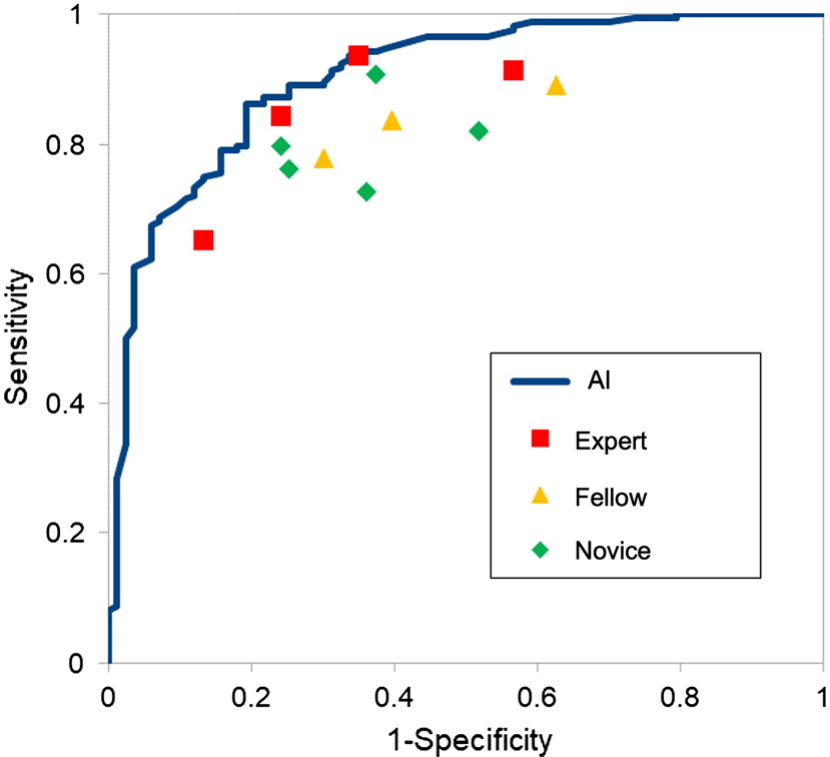
ROC curve of the trained model and diagnostic performance of endoscopists in the external validation. External validation with data from an observational study in humans demonstrated that the AI system had the same diagnostic sensitivity as expert endoscopists and was superior to that of fellows and novices

The sensitivity and specificity of the AI system for neoplastic lesion were 88.3% (95% CI 82.6–94.1%) and 90.3% (95% CI 83.0–97.7%), respectively (Table 3). The area under the receiver operating characteristic (ROC) curve was 0.903 (0.860–0.946). External validation with data from an observational study in humans demonstrated that the AI system had a diagnostic sensitivity comparable to that of expert endoscopists and superior to that of fellows and novices (Fig. 2). Specifically, the AI system had a diagnostic sensitivity comparable to that of expert endoscopists and better than fellows and novices, whereas the specificity exceeded that of all endoscopists (Table 3). The median sensitivity and specificity of all endoscopists were 85.0% (range 65.0–93.3%) and 69.4% (range 43.5–90.3%), respectively. Sensitivity was higher according to skill level: expert, 87.9% (range 65.0–93.3%); fellow, 85.0% (range 80.8–89.2%); and novice 82.5% (range 75.0–90.0%). Specificity was also higher according to skill level: expert, 72.6% (range 48.4–90.3%); fellow, 71.0% (range 50.0–82.3%); and novice, 66.1% (range 43.5–71.0%). The median inference speeds of the AI system and the endoscopist were 12.9 ms/image (interquartile range = 12.8–14 ms/image) and 1830 ms/image (interquartile range = 1660–2260 ms/image) to analyze the same set of 255 images (Table 3).

**Table 3:** External validation of the diagnostic performance of the AI system and endoscopists for predicting the pathology of early-stage colorectal cancers and precursor lesions

	All endoscopists	Experts	Fellows	Novice	AI
	*n* = 12	*n* = 4	*n* = 3	*n* = 5	
Sensitivity (median)	88.9%	89.6%	90.3%	84.7%	90.1%
IQR	84.7–90.6	83.3–92.4	88.2–90.3	84.7–90.3	NA
95% CIS	NA	NA	NA	NA	83.6–96.6
Specificity (median)	65.8%	67.1%	55.3%	68.4%	72.3%
IQR	53.9–76.3	53.3–78.9	47.4–61.8	63.2–76.3	NA
95% CIS	NA	NA	NA	NA	60.0–85.1
Inference time, [sec/image] (median)	1.83	1.72	2.17	1.86	0.0129ss
IQR	1.66–2.26	1.63–1.84	1.92–2.34	1.69–2.51	0.0128–0.014

The *AI* model and the endoscopists were tested using the same 255 images

*AI* artificial intelligence, *IQR* interquartile range, *NA*, not assessed

The sensitivity, specificity, and NPV in the WLI subgroup and in the IEE subgroup for the AI system and endoscopists are summarized in Supplementary Table 8 and the differences in performance according to endoscope manufacturers are summarized in Supplementary Table 9. Detailed information is provided in the online supplemental materials.

Among all 255 images, 28.6% (73 images) were diagnosed with a confidence score lower than 0.9 and excluded from the analysis. The distribution was high at 37% of the WLI images in the neoplastic lesions compared to 27% of the WLI images in the non-neoplastic lesions, and 22% of the IEE images in the neoplastic and non-neoplastic lesions.

### Visualizing the internal features of convolutional neural networks (CNNs) with t-SNE

**Fig. 3:**
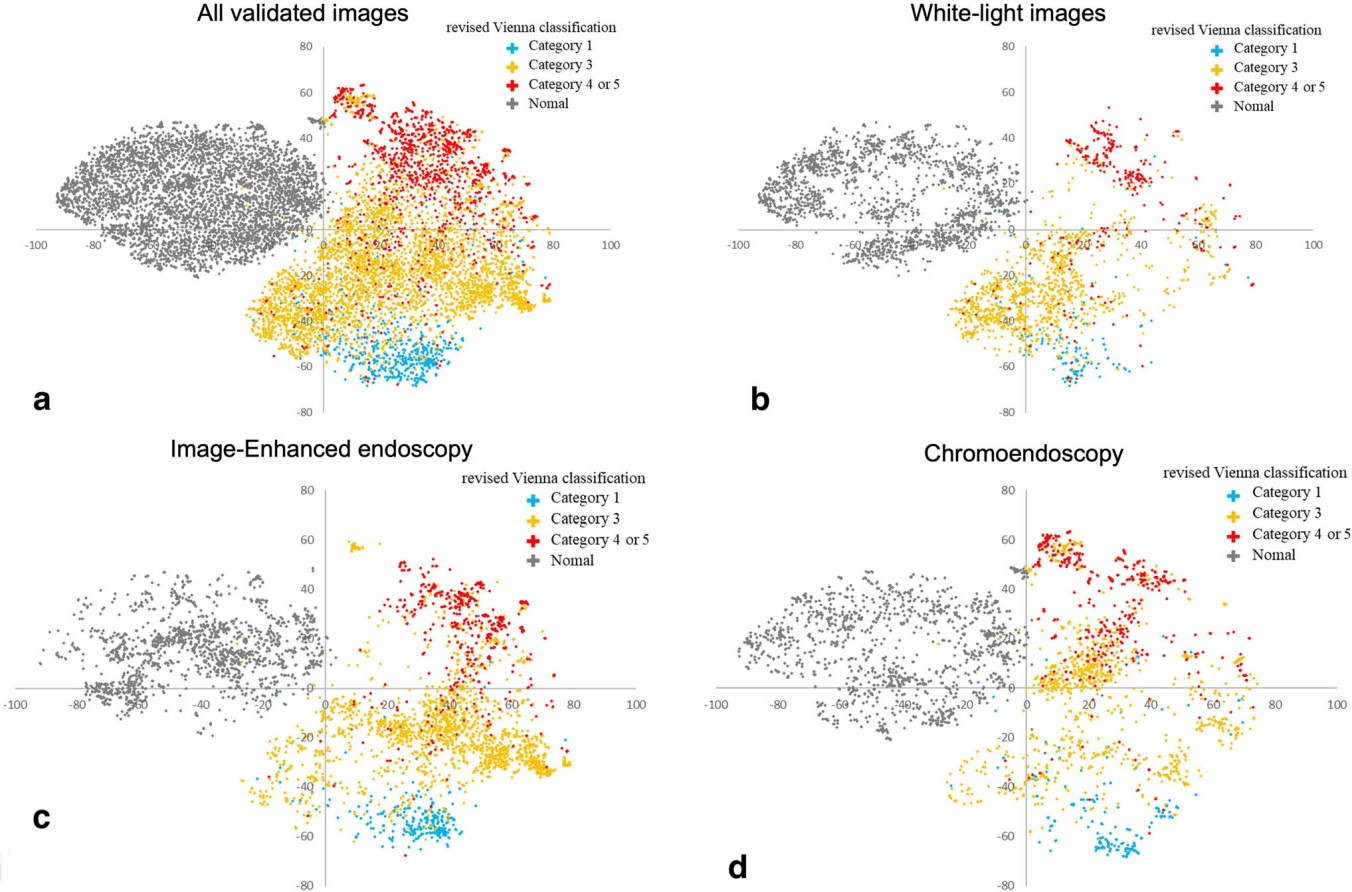
t-SNE visualization from the last hidden layer of the trained ResNet-152 for the four categories. All endoscopic images were marked with their internal features of the trained ResNet-152 and corresponding revised Vienna Classification. Most of the endoscopic images were well separated into the revised Vienna Classifications. **a** All validated images in the internal validation study. **b** White light images. **c** Image-enhanced endoscopy. **d** Chromoendoscopy

The internal features of the fully trained ResNet152 model are shown in Fig. 3. Each point represents the projection of the 2048-dimensional features of ResNet152 onto two dimensions for an endoscopy image. NA clusters and lesion clusters containing categories 1, 3, and 4/5 are clearly separated on the left and right, respectively, and lesion clusters are also well classified into the 3 categories. However, category 3 and category 4/5 are slightly mixed (Fig. 3a). In the WLI subgroup, the category 3 cluster is well separated from the category 4/5 cluster, whereas the category 1 and category 3 clusters overlap slightly (Fig. 3b). In the IEE subgroup, the category 1 and 3 clusters are more clearly separated than in the WLI subgroup (Fig. 3c), but there is a slight overlap between the category 3 cluster and the category 4/5 cluster. In the chromoendoscopy subgroup, the category 3 cluster overlaps with the category 4/5 cluster more than in the IEE subgroup (Fig. 3d).

## Discussion

In this study, we developed an AI system that uses deep learning techniques to automatically and robustly predict the pathological diagnosis of the revised Vienna Classification from standard and common endoscopic colonoscopy images. In recent years, attempts have been made to develop AI systems that can predict the pathological diagnosis of colorectal tumors by using deep learning and support vector machines, and some meaningful and valuable data have been reported [18, 22, 24–28]. Although improving the optical diagnosis of benign lesions can reduce pathological costs, models incapable of diagnosing high-grade tumors, including cancer, are unsatisfactory for clinical use due to the multi-step process of colorectal carcinogenesis. Furthermore, in order for the research results to be discussed worldwide, it is necessary to use globally uniform pathological diagnostic criteria for this type of research. Therefore, we adopted a multi-class classification for the output class of the CNN, which is based on the revised Vienna Classification, a widely known international classification method. In this study, we included four clinically important categories of the multi-class classification: category 1 (non-neoplastic lesion), category 3 (low-grade adenoma/dysplasia), and category 4 (high-grade adenoma/dysplasia) or 5 (invasive carcinoma). Each of these four classifications has different clinical treatment strategies, including observation, cold polypectomy, endoscopic mucosal resection, and surgery, so classifying them is a clinically important issue [29].

The strengths of our AI system include (1) high diagnostic performance based on approximately 70,000 images of more than 8500 unique lesions, (2) multi-class classification according to the Vienna Classification and the multi-step pathway of the colorectal carcinogenesis, (3) performance confirmed by internal and external validation, and (4) availability for use with non-magnified endoscopic images from multiple manufacturers. To the best of our knowledge, two recent studies have reported high diagnosis performance with the 3-classification AI model (CADx). Minegishi et al. reported a single-arm prospective study using CADx for narrow-band imaging (NBI-CADx) classifying 3-classes: hyperplastic polyp, sessile serrated lesion, and neoplastic/adenomatous. Using still NBI images, the NBI-CAD achieved an overall 93.3% sensitivity and 61.5% specificity for diagnosing diminutive (< 5 mm) neoplasms [30]. Similar results were reported by Ozawa et al. using about 17,000 images from about 4800 colorectal polyps and about 4000 normal colorectum images [31]. Although the sensitivity was high, the low specificity may to lead the misidentification of non-neoplasms as neoplasms, resulting in shorter surveillance intervals. Even including WLI images, the high sensitivity and specificity for diagnosing neoplastic lesions in external validation at 88.3 and 90.3% in our study suggest that a large amount of training data, about 57,000 images from about 7000 unique lesions, may have affected the performance, and this AI system can be used to provide precise feedback to physicians in clinical settings.

We have developed a robust, high-performance diagnostic model that aims to enable the use of AI during colonoscopy without interfering with the physicians’ operations. The accuracy of discriminating between neoplasms and non-neoplasms using WLI is reported to be about 70% sensitivity and about 80% specificity in both experts and endoscopists in general hospitals. The AI system developed in this study achieved a sensitivity of 70–80% and specificity of almost 100% in all four categories, suggesting that high diagnostic accuracy was achieved even for WLI. Although previous studies have reported that expert endoscopists show high sensitivity (about 85–95%) in optical diagnosis using IEE and pit pattern, specificity is only about 70–80%, and not all community-based gastroenterologists show such high performance [11, 32, 33]. Our AI system achieved > 80% sensitivity and almost 100% specificity in all four categories in IEE. Interestingly, for all endoscopic images in the internal validation set, we applied t-SNE to visualize the internal features of ResNet152 based on the revised Vienna Classification. The endoscopic images were well classified by the internal features into different clusters according to the three categories of the revised Vienna Classification and NA, particularly in IEE. Therefore, the diagnostic performance of the developed AI model is considered to be comparable to that of experts, such as endoscopists at university hospitals and high-volume centers.

WLI, an optical digital image such as NBI/BLI, and occasional use of chromoendoscopy are sequentially used for one lesion in a clinical setting. In the AI system, the sensitivity for diagnosing category 1 in IEE (NBI/BLI) increased about 6%, decreased about 4% in category 3, and increased about 11% in category 4 or 5, compared to WLI. In chromoendoscopy, the sensitivity for diagnosing category 4 or 5 increased by about 13% than WLI (Supplementary Table 7). In addition, the sensitivity for diagnosing neoplastic lesion was the same between the WLI and IEE (NBI/BLI), but specificity was about 6% higher in IEE than WLI in the external validation (Supplementary Table 8). Therefore, it is considered that IEE has an additional effect over the WLI in diagnosing HP or SSL, and chromoendoscopy has an additional effect on narrowing the diagnosis of high-grade dysplasia or cancer.

In addition, the principal aim of this AI system was to prevent misdiagnosis by endoscopists in colonoscopy; therefore, we confirmed the superiority of the sensitivity and specificity by comparing the performance of the system with that of various endoscopists. Previous studies on computer-assisted diagnosis (CADx) have reported extremely high diagnostic performance; however, neoplastic/non-neoplastic classification was the main outcome and no studies were confirmed by external validation. Therefore, we performed external validation to verify the ability of our AI model to discriminate neoplastic/non-neoplastic classification and to compare its performance with various endoscopists, following these previous studies. Using endoscopic images of conventional observation from seven other institutions, the present AI model was confirmed to achieve a sensitivity of 88.3% and specificity of 90.3%. The observational study demonstrated that this AI system has a diagnostic sensitivity comparable to that of endoscopy experts and is superior to that of fellows and novices. In terms of expert performance using IEE, the sensitivity was above 90%, exceeding the 87.9% achieved by the AI system, but the specificity was only 70%, which is lower than the 93.1% achieved by the AI system. Although high sensitivity and specificity are strengths of this AI system, it was found that when the prevalence of neoplastic lesions was about 67%, its NPVs were about 80% for both WLI and IEE, missing the threshold of 90% laid out in the Preservation and Incorporation of Valuable endoscopic Innovations (PIVI) statement. Because 19% (49/255 images) of the images in the external validation set were of tumors 10 mm or larger, the NPV was not considered to exceed 90% (Supplementary Table 3). Interestingly, although the endoscopist took about 2 s to judge each image, the inference time of the AI system was only 0.01 s and the AI model was successfully implemented in colonoscopy videos (Supplementary Video 1). Therefore, when used in combination with the previously developed lesion detection method, our system is capable of performing all tasks, from lesion detection to diagnosis [7].

We reviewed all images of the lesion that AI made a misdiagnosis in the external validation and found there were two reasons: (1) lesion itself was difficult to diagnose, (2) lesion was not properly visualized, such as out of focus, etc. These images were considered difficult to diagnose even by endoscopists. Therefore, even if AI diagnostic support became widespread, it is considered that endoscopists would need to acquire endoscopic skills so that they could capture images in which lesions were captured center with proper focus.

This study has some limitations. First, the major limitation was its retrospective design and the distribution of the images with a lower than 0.9 confidence score, 13.2% in the internal validation and 28.6% in the external validation, is expected to be higher in clinical practice. Therefore, it is considered that multiple images are necessary for AI diagnosis in clinical practice AI diagnosis support. Second, although this AI model predicts four clinically important categories (the three categories of the revised Vienna Classification and normal mucosa), it cannot discriminate high-grade dysplasia and invasive cancer (categories 4 and 5 of the revised Vienna Classification) due to the relatively small number of invasive cancers in our datasets. In addition, 89% (2849/3196 images) of the Chromoendoscopy data were indigo carmine dye spraying images, while the number of crystal violet staining images were small at 347/3196 images (11%) and most of the images were category 4 or 5 (Supplementary Table 7). Further study is needed to classify category 5 of the revised Vienna Classification separately. Third, the external validation set in the present study was limited to the distinction between neoplastic/non-neoplastic lesions due to the small number of images/lesions, so we cannot perform a validation of the system using multi-class classification. Also, since most of the lesions in the external validation were small lesions < 10 mm, chromoendoscopy images could not be collected (Supplementary Table 4). In addition, pathological diagnoses were not centrally reviewed in the external validation. A further large-scale external validation study is needed to address this issue. Fourth, Images with and without magnification were included, however, we were unable to analyze the diagnostic performance separately due to a lack of supplemental information about magnification. However, an advantage of this AI system is that it can be applied to many of the endoscopes developed by Japan’s two major manufacturers, Olympus Medical and Fujifilm Medical. Thus, this AI model is considered more robust than other reported systems. We are planning to start in vivo clinical trials using this AI system.

In conclusion, we have developed an AI system that automatically predicts the revised Vienna Classification of CRC in colonoscopy. The present results suggest that this AI system can support endoscopists to avoid misdiagnosis, thereby improving the differential diagnosis of colorectal cancer.

## Supplementary Information

Below is the link to the electronic supplementary material.

Supplementary file1 (DOCX 108 KB)

Supplementary file2 (TIFF 9950 KB)

Supplementary file3 (TIFF 25774 KB)

Supplementary file4 (TIFF 184 KB)

Supplementary file5 (TIFF 2971 KB)

Supplementary file6 (TIFF 250 KB)

Supplementary file7 (TIFF 394 KB)

Supplementary file8 (TIFF 1295 KB)

Supplementary file9 (MP4 64769 KB)

## Abbreviations

AI: Artificial intelligence
GPUs: Graphics processing units
CRC: Colorectal cancer
IEE: Image-enhanced endoscopy
WLI: White light imaging
HP: Hyperplastic polyp
SSL: Sessile serrated lesion
NA: Normal image
NBI: Narrow-band imaging
BLI: Blue laser imaging
DA: Data augmentation
ROIs: Regions of interest
PPV: Positive predictive value
NPV: Negative predictive value
95% CI: 95% Confidence interval
t-SNE: T-distributed stochastic neighbor embedding
PIVI: Preservation and Incorporation of Valuable endoscopic Innovations
