# Supplementary material for: Robust automated prediction of the revised Vienna Classification in colonoscopy using deep learning: development and initial external validation
Source: J Gastroenterol. 2022 Aug 16;57(11):879–89. doi: 10.1007/s00535-022-01908-1 (PMC9596523; doi:10.1007/s00535-022-01908-1)
Supplement: Supplementary file 1 — Supplementary file1 (DOCX 108 KB) [file 535_2022_1908_MOESM1_ESM.docx]

Supplementary Information for

**Robust** **automated prediction of revised Vienna Classification in colonoscopy using deep learning: a development and external validation study**

Masayoshi Yamada, Ryosaku Shino, Hiroko Kondo, Shigemi Yamada,
Hiroyuki Takamaru, Taku Sakamoto, Pradeep Bhandari, Hitoshi Imaoka, Aya Kuchiba,
Taro Shibata, Yutaka Saito, Ryuji Hamamoto

**Contents**

- Supplementary methods
  - Building deep learning algorithms
  - T-distributed stochastic neighbor embedding (t-SNE) analysis
  - Statistical analysis
- Supplementary results
  - Characteristics of lesions
  - Deep learning algorithm
  - Diagnostic performance of the AI system in the internal validation test
  - External validation of AI performance in differentiating neoplastic and non-neoplastic lesions and comparison with endoscopists
- Discussion for selection of Deep learning algorithm
- Supplementary references
- Supplementary figure legends
  - Supplementary Fig. 1 Representative images in image-enhanced endoscopy
  - Supplementary Fig. 2 Representative images in chromoendoscopy
  - Supplementary Fig. 3 Residual block (bottleneck) of the ResNet152 architecture
  - Supplementary Fig. 4 Representative images in data augmentation
  - Supplementary Fig. 5 AlexNet architecture
  - Supplementary Fig. 6 MBConv [N] architecture
  - Supplementary Fig. 7 Sensitivity and specificity of the trained models on the internal validation set
  - Supplementary Video 1 Representative colonoscopy video images using the developed AI system for automated prediction of revised Vienna Classification in colonoscopy
- Supplementary Tables
  - Supplementary Table 1 Clinicopathological characteristics of lesions
  - Supplementary Table 2 ResNet152 architecture
  - Supplementary Table 3 Number of images used for deep learning training and validations stratified by tumor location, size, and morphology
  - Supplementary Table 4 Details of the images used in the external validation test
  - Supplementary Table 5 Sensitivities stratified by tumor location, size, and morphology in the internal validation
  - Supplementary Table 6 The distribution of the type of polyps classified the AI system in the internal validation
  - Supplementary Table 7 Diagnostic performance stratified by modality and manufacturer in the internal validation
  - Supplementary Table 8 Diagnostic performance of the AI system for predicting the pathology of early-stage colorectal cancers and precursor lesions stratified by modality in the external validation
  - Supplementary Table 9 Diagnostic performance of the AI system for predicting the pathology of early-stage colorectal cancers and precursor lesions stratified by endoscope manufacturer in the external validation
  - Supplementary Table 10 EfficientNet-B8 architecture

**Supplementary methods**

***Building deep learning algorithms***

Three types of deep learning algorithms (AlexNet, ResNet152, and EfficientNet-B8) were trained to learn colonoscopy features of diseases in order to compare neural networks with small and large numbers of layers (Supplementary Figs. 3,5 and 6, Supplementary Tables 2 and 10)^1-3^. Our learning process was single shot (i.e., a one-time training process).

In the preliminary study, the ImageNet Pretrained-model showed higher performance compared with scratch training, so training was performed using a pretrained model with stochastic gradient descent. The number of batches, learning rate, number of iterations, and momentum were 20, 1e-3, 67,368, and 0.9, respectively. Calculations were run on the graphics processing unit (NVIDIA GeForce RTX 2070) of a personal computer. All lesions were manually circumscribed by a rectangle, cropped for training, and converted to a resolution of 256 × 256 pixels. Preliminary results indicated that the accuracy of the trained model was better with data augmentation (DA) than without DA (0.557 vs. 0.462).

DA using JPG recompression (re-encoding with quality), hue manipulation (randomly changing the hue value in the hsv space), brightness manipulation (adding brightness in the hsv space), blur (adds blur to up to 3 areas of 100 × 100 pixels), and masking (adds Gaussian noise to up to 3 areas of 100 × 100 pixels) were used to eliminate the class imbalance between the four categories (Supplementary Fig. 4) ^4^.

***T-distributed stochastic neighbor embedding (t-SNE) analysis***

We analyzed the internal features of the fully trained ResNet152 model by t-SNE analysis. The 2,048-dimensional features of ResNet152 were projected into two dimensions and visualized in different colors for each category, by processing the internal validation data with T-SNE, which is a dimension-reduction method proposed by van der Maaten and Hinton in 2008 ^5^ that is characterized by non-linear mapping in a low-dimensional space such that sets with high similarity are close together and sets with low similarity are far apart. The algorithm of the t-SNE consists mainly of two stages. First, a probability distribution is constructed so that similar sets are likely to be selected for each pair of high-dimensional data, while the probability distribution is very small. Second, a similar probability distribution is defined for a set on a low-dimensional map and find the location of points in a low-dimensional map that minimizes the amount of Calback Liveer information between the two distributions. Unlike rudimentary dimensional reduction methods such as PCA, t-SNE has the advantage of dimensional reduction even in relationships that cannot be expressed by linearity.

***Statistical analysis***

The diagnostic performance of the trained model was evaluated by estimating the sensitivity, specificity, NPV, and PPV with their Clopper–Pearson exact 95% CIs. Overall sensitivity was calculated as the total number correctly predicted as categories 1, 3, and 4 or 5 by the AI divided by the total number of ground truth (GT) images in these categories. Overall specificity was calculated as the number correctly predicted as NA by the AI divided by number of GT NA. Overall PPV was calculated as the total number of GT images in categories 1, 3, and 4 or 5 divided by the number of images predicted as categories 1, 3, and 4 or 5 by the AI. Overall NPV was calculated as the number of GT NA divided by the number of images predicted as NA by the AI. Overall accuracy was calculated as the total number of images correctly identified as categories 1, 3, and 4 or 5 and NA by the AI divided by the total number of images. In the internal validation, accuracy, PPV, and NPV were calculated with an assumption that the ratio of lesions (including category 1, 3, 4 and 5) to non-lesions (including category NA) as 60:40.

In the external validation, the diagnostic performance was evaluated by lesion-based rather than the image-based following a clinical practice. Regarding the definition of diagnostic accuracy when there were multiple images in the same lesion, AI calculates the sensitivity and specificity by the majority decision of the image with a confidence score of 0.9 or higher images. If AI showed different results for multiple images of the same lesion (if it is not decided by a majority decision), the neoplastic lesion was selected as the AI diagnosis. Regarding endoscopists, sensitivity and specificity were calculated by a majority decision on each image's diagnostic results. If it is not decided by a majority decision, the neoplastic lesion was selected as the endoscopists’ diagnosis, as in the AI calculation method. We also calculated an ROC curve and the area under the curve (AUC) based on the different cutoffs of confidence scores of the trained model for each image with neoplastic or non-neoplastic lesions.

**Supplementary results**

***Characteristics of lesions***

The clinicopathological characteristics of the lesions in the validation sets are shown in Table 1. About 50%–60% of the lesions were located at the right-sided colon, and the median size of the lesions was about 5 mm in diameter. In the internal validation set, 30.8% of the lesions were the polypoid type and 66.2% were the slightly elevated or depressed type. In the external validation set, 44.5% of the lesions were the polypoid type and 55.5% were the slightly elevated or depressed type. Lesions falling into categories 1, 3, 4, 5.1, and 5.2 in the revised Vienna Classification accounted for 11.5%, 76.6%, 10.0%, 1.6%, and 0.3% of lesions in the internal validation set, respectively. In the external validation set, 36.7% of lesions were non-neoplastic lesions (category 1), and 63.3% were neoplastic lesions. The numbers of images used for internal and external validation for each modality (WLI, IEE, and chromoendoscopy) are summarized in Table 2. The number of images in category 1 was lower than that in other categories, especially WLI and chromoendoscopy. The number of images stratified by tumor location, tumor size, and morphology is summarized in Supplementary Table 3. The number of images was low for polypoid morphologies in category 1 and tumors less than or equal to 5 mm in size in category 4 or 5. In addition, Supplementary Table 4 summarizes the details of the manufacturer and modality (WLI or IEE) used in the external verification set; the prevalence of neoplastic lesions was 67.5%, and 77.3% of the images were taken with Olympus endoscopes.

***Deep learning algorithm***

The sensitivity and specificity in the internal validation set were respectively 70.8% and 93.1% in category 1, 71.1% and 91.1% in category 3, and 68.6% and 93.1% in category 4 or 5 in AlexNet; 73.9% and 96.7% in category 1, 79.5% and 92.0% in category 3, and 73.2% and 93.2% in category 4 or 5 in ResNet152; 77.2% and 96.5% in category 1, 71.6% and 92.4% in category 3, and 71.6% and 92.7% in category 4 or 5 in EfficientNet-B8 (Supplementary Fig. 7). Mean inference time of AlexNet, ResNet152, and Efficient-B8 were 0.5603 ms/image, 13.78 ms/image, 27.45 ms/image, respectively.

***Diagnostic performance of the AI system in the internal validation test***

The raw data show that of the 5,427 images that the AI system identified as normal, only one was category 4 or 5 (Supplementary Table 6). In the subgroup analysis by modality, location, and size, the sensitivities of category 4 or 5 in WLI, categories 1 and 3 in chromoendoscopy, and category 4 or 5 when using the Fujifilm endoscope were relatively low at 71.1% (95% CI: 66.3%–75.8%), 76.7% (95% CI: 71.1%–82.3%), 78.4% (95% CI: 75.9%–80.8%), and 68.5% (95% CI: 61.0%–76.0%), respectively (Supplementary Table 7). In the subgroup analysis of sensitivities by tumor location, tumor size, and morphology, the left-sided colon was less sensitive than the right-sided colon in categories 1 and 3 (86.2% vs. 76.9% and 88.4% vs. 80.2%), and the right-sided colon was less sensitive than the left-sided colon in categories 4 or 5 (75.7% vs. 82.4%) (Supplementary Table 5). Regarding tumor size, in category 1, the sensitivity of the ≤5 mm group was 73.0%, which was lower than the other categories (Supplementary Table 5). The sensitivities of the ≥10 mm group in category 3, the ≤5 mm group, and the ≥6, ≤9 mm group in category 4 or 5 were low at 69.3%, 31.6%, and 46.8%, respectively (Supplementary Table 5). In terms of morphology, in categories 1 and 3, the sensitivity of the polypoid type was lower than that of the slightly elevated or depressed types (75.3% vs. 83.7% and 79.0% vs. 88.2%, respectively). In contrast, in category 4 or 5, the sensitivity of the polyp type was higher than that of the slightly elevated or depressed type (86.0% vs. 62.0%) (Table 4).

***External validation of AI performance in differentiating neoplastic and non-neoplastic lesions and comparison with endoscopists***

In the subgroup analysis, the sensitivity, specificity, and NPV of the AI system were respectively 88.7% (95% CI: 80.8%–96.6%), 87.9% (95% CI: 80.8%–96.6%), and 80.6% (95% CI: 67.6%–93.5%) in the WLI subgroup and 87.9% (95% CI: 79.5%–96.3%), 93.1% (95% CI: 83.9%–100%), and 79.4% (95% CI: 65.8%–93.0%) in the IEE subgroup (Supplementary Table 8). In the expert endoscopists, the sensitivity, specificity, and NPV were 84.7% (95% CI: 62.9%–90.3%), 71.2% (95% CI: 57.6%–90.9%), and 69.9% (95% CI: 56.6%–78.6%) in the WLI subgroup, compared with 91.4% (95% CI: 67.2%–96.6%), 74.1% (95% CI: 37.9%–89.7%), and 78.0% (95% CI: 57.8–90.5%) in the IEE subgroup (Supplementary Table 8). Furthermore, we also examined the differences in performance between endoscope manufacturers (Supplementary Table 9). The sensitivity, specificity, and NPV of the AI system were 100% (95% CI: 100%–100%), 93.3% (95% CI: 80.7%–100%), and 100% (100%–100%) in the Fujifilm subgroup and 85.1% (95% CI: 77.9%–92.3%), 89.4% (95% CI: 80.5%–98.2%), and 75.0% (95% CI: 63.7%–86.3%) in the Olympus subgroup.

**Discussion for selection of Deep learning algorithm**

Another expected benefit of this study is the identification of suitable deep learning algorithms for colonoscopy images. In the present study, three types of deep learning algorithms (AlexNet, ResNet152, and EfficientNet-B8) were trained to detect the features of the diseases screened by colonoscopy.

AlexNet is a network proposed by Krizhevsky *et al*. that achieved state-of-the-art (SOTA) performance in the ImageNet Large Scale Visual Recognition Challenge (ILSVRC) in 2012^1^. Compared with ResNet and EfficientNet, it has fewer layers and is lighter (Supplementary Fig. 5).

ResNet is a network proposed by He *et al*. in 2015, which achieved state-of-the-art performance in ILSVRC 2015 and can process up to 152 layers ^2^. ResNet comprises multiple layers of residual blocks, as shown in Supplementary Fig. 3. ResNet152 has layers that are 19 times deeper than those of AlexNet but does not suffer from the degradation problem caused by vanishing gradients due to residual blocks, which is a bottleneck, and thus stable learning is expected (Supplementary Fig. 3 and Supplementary Table 2). The residual block is characterized by a structure called a “skip connection” that skips layers, thereby enabling stable learning without vanishing gradients, even when multiple blocks are stacked. Supplementary Table 2 shows the configuration of ResNet152 employed in the present study.

EfficientNet is a deep learning model proposed by Tan *et al*. in 2019 and also achieved state-of-the-art performance in the ILSVRC^3^. This model was developed by tuning the depth, width, and resolution of each layer with neural architecture search (NAS) based on the MBConv block and obtains high accuracy with a small number of parameters. The MBConv [N] block is the Inverted Residuals and Linear Bottlenecks block used in MobileNet V2, a lightweight network, with the Squeeze-and-Excitation (SE) module, which was introduced to improve accuracy. Supplementary Fig. 6 shows the details of MBConv, where [N] indicates how many times the number of channels is expanded in the first convolution layer. Supplementary Table 10 shows the configuration of Efficient-B8 used in the present study.

The diagnostic performance of AlexNet with fewer layers was lower than that of EfficientNet-B8 and ResNet152 with more layers. EfficientNet-B8, which has more layers than ResNet152, was slightly inferior to ResNet152. The inference time of AlexNet, which has a small number of layers, was the shortest among the three algorithms, whereas the inference time of EfficientNet-B8 was about twice as long as that of ResNet152. Therefore, if the number of layers is comparable to that of ResNet152, the classification performance can be guaranteed. However, increasing the number of layers beyond ResNet152 does not improve performance and only increases the inference time. On the basis of these results, we chose ResNet152 as the prediction model because it had the highest accuracy and fastest inference time.

Supplementary References

1 Krizhevsky, A., Sutskever, I. & Hinton, G. E. Imagenet classification with deep convolutional neural networks. *Adv. Neural Inf. Process. Syst.* **25**, 1097-1105 (2012).

2 He, K., Zhang, X., Ren, S. & Sun, J. Deep Residual Learning for Image Recognition. *2016 IEEE Conference on Computer Vision and Pattern Recognition (CVPR)*, 770-778, doi:10.1109/CVPR.2016.90 (2016).

3 Tan, M. & Le, Q. in *International Conference on Machine Learning.* 6105-6114 (PMLR).

4 Zhong, Z., Zheng, L., Kang, G., Li, S. & Yang, Y. in *Proceedings of the AAAI Conference on Artificial Intelligence.* 13001-13008.

5 Van der Maaten, L. & Hinton, G. Visualizing data using t-SNE. *Journal of machine learning research* **9** (2008).

**Supplementary figure legends**

**Supplementary Figure 1: Representative images in image-enhanced endoscopy. a, e, i** Superficial. **b, f, j** Protruded. **c, g, k** Laterally spreading tumor type. **d, h, i** Flat and depressed type. **a, b, c, d** Category 1 in the revised Vienna Classification. **e, f, g, h** Category 3 in the revised Vienna Classification. **i, j, k, l** Category 4 or 5 in the revised Vienna Classification.

**Supplementary Figure 2: Representative images in chromoendoscopy. a, e, i** Superficial. **b, f, j** Protruded. **c, g, k** Laterally spreading tumor type. **d, h, i** Flat and depressed type. **a, b, c, d** Category 1 in the revised Vienna Classification. **e, f, g, h** Category 3 in the revised Vienna Classification. **i, j, k, l** Category 4 or 5 in the revised Vienna Classification.

**Supplementary Figure 3: Residual block (bottleneck) of the ResNet152 architecture.** Conv: Convolution layer.

**Supplementary Figure 4: Representative images in data augmentation. a** Original endoscopic image. **b** JPG recompression. **c** Hue manipulation. **d** Brightness change. **e** Blur. **f** Mask.

**Supplementary Figure 5: AlexNet architecture.** Conv: Convolution layer. Activation: Activation function. MaxPooling: MaxPooling layer. FC: Fully connected layer.

**Supplementary Figure 6: MBConv[N] architecture.** Conv: Convolution layer. Batch Norm: Batch normalization. Activation: Activation function. Depthwise Conv: Depthwise convolution layer. Global Pooling: Global average pooling layer. FC: Fully connected layer. SE Block: Squeeze and excitation block.

**Supplementary Figure 7: Sensitivity and specificity of the trained models in the internal validation set.** Categories 1, 3, and 4 or 5 are based on the revised Vienna Classification.

**Supplementary Video 1:** **Representative colonoscopy video images using the developed AI system for automated prediction of the revised Vienna Classification in colonoscopy.**

**Supplementary Table 1:** **Clinicopathological characteristics of lesions**

|  | **Training** | **Hyperparameter tuning** | **Internal**  **validation** | **External**  **validation** |
| --- | --- | --- | --- | --- |
| Number of lesions, n | 5650 | 1125 | 1718 | 128 |
| Location of lesions*, n  Right-sided colon  Left-sided colon  Rectum | 3138 (55.5%)  1952 (34.5%)  560 (9.9%) | 611 (54.3%)  384 (34.1%)  130 (11.6%) | 1035 (60.2%)  556 (32.4%)  127 (7.4%) | 66 (51.6%)  53 (41.4%)  9 (7.0%) |
| Size of lesions, mm, median (IQR)  (range) | 5 (4–8)  (1–70) | 6 (4–10)  (1–60) | 5 (4–8)  (1–80) | 5 (4–8)  (2–24) |
| Morphological type^†^, n (%)  Polypoid  Slightly elevated and depressed  LST-G  LST-NG  Recurrent | 1966 (34.8%)  3425 (60.6%)  89 (1.6%)  167 (3.0%)  3 (0.1%) | 367 (32.6%)  694 (61.7%)  17 (1.5%)  45 (4.0%)  2 (0.2%) | 529 (30.8%)  1137 (66.2%)  14 (0.8%)  37 (2.2%)  1 (0.1%) | 57 (44.5%)  71 (55.5%)  0  0  0 |
| Revised Vienna Classification, n (%)  Category  1 Negative for neoplasia/dysplasia  Hyperplastic polyp  Sessile serrated lesion  Serrated lesion^¶^  3 Low grade adenoma/dysplasia  4.1 High-grade adenoma/dysplasia  5.1 Intramucosal carcinoma  5.2 Submucosal carcinoma or beyond | 1086 (19.2%)  475 (8.4%)  600 (10.6%)  11 (0.2%)  3489 (61.8%)  908 (16.1%)  122 (2.2%)  45 (0.8%) | 274 (24.4%)  113 (10.0%)  160 (14.2%)  1 (0.1%)  585 (52.0%)  218 (19.4%)  27 (2.4%)  21 (1.9%) | 198 (11.5%)  79 (4.6%)  117 (6.8%)  2 (0.1%)  1316 (76.6%)  171 (10.0%)  28 (1.6%)  5 (0.3%) | 47 (36.7%)  30 (23.4%)  17 (13.3%)  0  81 (63.3%) |
| Normal^§^, images | 11056 | 2805 | 5491 | 0 |

*, Right-sided colon includes the cecum, ascending colon and transverse colon; Left-sided colon includes the descending colon and sigmoid colon; Rectum includes the rectosigmoid colon and upper and lower rectum. †, Polypoid type includes 0-Is, Isp, Ip; Slightly elevated and depressed includes 0-IIa, IIb, IIc; LST-G, granular type laterally spreading tumor; LST-NG, non-granular type laterally spreading tumor. ¶, Serrated lesions which are difficult for discriminate SSL from HP. §, Images without lesions. IQR, interquartile range.

**Supplementary Table 2: ResNet152 architecture**

| **Stage** | **Operator** | **Resolution** | **Layers** |
| --- | --- | --- | --- |
| 1 | Conv 7×7, 64 | 128 | 1 |
| 2 | Conv 3×3 | 64 | 1 |
| 3 | 1×1 64  3×3 64  1×1 256 | 64 | 3 |
| 4 | 1×1 128  3×3 128  1×1 512 | 32 | 8 |
| 5 | 1×1 256  3×3 256  1×1 1024 | 16 | 36 |
| 6 | 1×1 512  3×3 512  1×1 2048 | 8 | 3 |
| 7 | FC 4 | - | 1 |

**Supplementary Table 3: Number of images used for deep learning training and validations stratified by tumor location, size, and morphology**

| **Revised Vienna Classification** | **Training** | **Hyperparameter**  **tuning** | **Internal**  **validation** | **External**  **validation** |
| --- | --- | --- | --- | --- |
| Category 1*, images (lesions)  Tumor location  right side  left side and rectum  Tumor size  ≤5 mm  ≥6 mm, ≤9 mm  ≥10 mm  Morphology  polypoid  superficial | 7004 (1086)  4398 (618)  2606 (468)  2203 (465)  1979 (320)  2822 (301)  1261 (207)  5463 (849) | 1869 (274)  1212 (159)  657 (115)  537 (111)  601 (85)  731 (78)  359 (54)  1402 (209) | 963 (198)  595 (119)  368 (79)  303 (74)  273 (59)  387 (65)  133 (27)  830 (171) | 83 (47)  48 (27)  35 (20)  58 (30)  12 (8)  13 (9)  32 (17)  51 (30) |
| Category 3^†^, images (lesions)  Tumor location  right side  left side and rectum  Tumor size  ≤5 mm  ≥6 mm, ≤9 mm  ≥10 mm  Morphology  polypoid  superficial | 15693 (3489)  8957 (2060)  6736 (1429)  9199 (2409)  4371 (810)  2133 (270)  5069 (1080)  9751 (2301) | 2652 (585)  1456 (336)  1196 (249)  1565 (402)  725 (143)  362 (40)  770 (155)  1720 (413) | 5639 (1316)  3259 (826)  2380 (490)  3440 (913)  1360 (296)  839 (107)  1770 (375)  3662 (914) | 172 (81)  83 (40)  89 (41)  95 (46)  41 (19)  36 (16)  87 (39)  85(42) |
| Category 4 or 5^‡^, images (lesions)  Tumor location  right side  left side and rectum  Tumor size  ≤5 mm  ≥6 mm, ≤9 mm  ≥10 mm  Morphology  polypoid  superficial | 12629 (1075)  4517 (460)  8112 (615)  1018 (144)  2646 (283)  8965 (648)  7063 (679)  3521 (275) | 3146 (266)  1084 (116)  2080 (150)  208 (37)  612 (58)  2344 (171)  1687 (158)  865 (72) | 1955 (204)  748 (90)  1207 (114)  91 (17)  271 (45)  1593 (142)  1141 (127)  465 (52) |  |
| Normal, n (%)^§^ | 11056 | 2805 | 5491 | 0 |

*, Vienna classification Category 1 includes hyperplastic polyp or sessile serrated lesion; †, Category 3 includes low-grade adenoma/dysplasia; ‡, Category 4 or 5 included high-grade adenoma/dysplasia or submucosal invasive cancer; §, Images without lesion.

**Supplementary Table 4: Details of the images used in the external validation test**

|  |  | **WLI** | | **IEE (BLI/NBI)** | | **Total** | **%** |
| --- | --- | --- | --- | --- | --- | --- | --- |
|  |  | **n (lesion)** | **%** | **n (lesion)** | **%** | **n (lesion)** |  |
| Number of images (lesion) | | 144 (94) | 100 | 111 (105) | 100 | 255 (199^§^) |  |
| Manufacturer | Olympus | 120 (76) | 83.3 | 77 (74) | 69.4 | 197 (150) | 77.3 |
|  | Fujifilm | 24 (18) | 16.7 | 34 (31) | 30.6 | 58 (49) | 22.7 |
| Histology | Neoplastic* | 98 (60) | 68.1 | 74 (71) | 66.7 | 172 (131) | 67.5 |
|  | Non-neoplastic^†^ | 46 (34) | 31.9 | 37 (34) | 33.3 | 83 (68) | 32.5 |
|  | Hyperplastic | 36 (27) | 25.0 | 20 (19) | 18.0 | 56 (46) | 22.0 |
|  | SSL | 10 (7) | 6.9 | 17 (15) | 15.3 | 27 (22) | 10.6 |
| Tumor size | ≤5 mm | 90 (61) | 62.5 | 63 (59) | 56.8 | 153 (120) | 60.0 |
|  | ≥6 mm, ≤9 mm | 29 (21) | 20.1 | 24 (23) | 21.6 | 53 (44) | 20.8 |
|  | ≥10 mm | 25 (12) | 17.4 | 24 (23) | 21.6 | 49 (35) | 19.2 |
| Morphology | Polypoid | 73 (43) | 50.7 | 46 (46) | 41.4 | 119 (89) | 46.7 |
|  | Superficial | 71 (51) | 49.3 | 65 (59) | 58.6 | 136 (110) | 53.3 |

*, Category 3 or 4 in the Vienna Classification. †, Category 1 in the Vienna Classification. §, The 71 lesions captured using both WLI and IEE images and were counted in both groups. WLI, white-light imaging; IEE, image-enhanced endoscopy; BLI, blue laser imaging; NBI, narrow-band imaging; SSL, sessile serrated lesion.

**Supplementary Table** **5: Sensitivities stratified by tumor location, size, and morphology in the internal validation**

| **Revised Vienna Classification** | **Tumor location** | | **Tumor size** | | | | **Tumor morphology** | |
| --- | --- | --- | --- | --- | --- | --- | --- | --- |
|  | **Right-sided colon**  **%, (95% CIs)** | **Left-sided colon and Rectum**  **%, (95% CIs)** | **≤5 mm**  **%,**  **(95% CIs)** | **≥6, ≤9 mm**  **%,**  **(95% CIs)** | **≥10, ≤19 mm**  **%,**  **(95% CIs)** | **≥20 mm**  **%,**  **(95% CIs)** | **Polypoid**  **%, (95% CIs)** | **Slightly elevated and depressed**  **%, (95% CIs)** |
| Category 1^*^, images | 86.2  (83.0–89.3) | 76.9  (71.9–81.8) | 73.0  (67.3–78.8) | 86.2  (81.7–90.8) | 87.8  (83.9–91.7) | 84.8  (72.6–97.1) | 75.3  (66.5–84.0) | 83.7  (80.9–86.6) |
| Category 3^†^, images | 88.4  (87.1–89.6) | 80.2  (78.4–82.0) | 86.9  (85.7–88.2) | 88.5  (86.6–90.5) | 69.5  (65.7–73.2) | 63.2  (41.5–84.8) | 79.0  (76.9–81.1) | 88.2  (87.0–89.4) |
| Category 4 or 5^‡^, images | 75.7  (72.1–79.2) | 82.4  (79.9–84.8) | 31.6  (21.4–41.9) | 46.8  (39.3–54.3) | 87.5  (85.5–89.6) | 86.8  (82.5–91.0) | 86.0  (83.7–88.2) | 62.0  (56.9–67.2) |

*, Vienna Classification Category 1 includes hyperplastic polyp (HP) or sessile serrated lesion (SSL); †, Category 3 includes low-grade adenoma/dysplasia; ‡, Category 4 or 5 includes high-grade adenoma/dysplasia or submucosal invasive cancer; CI, confidence interval.

**Supplementary Table 6:** **The distribution of the type of polyps classified the AI system in the internal validation**

| **Revised Vienna Classification** | | **Ground Truth** | | | | **Total**  **n=12193** |
| --- | --- | --- | --- | --- | --- | --- |
|  |  | **Category 1^*^**  **n=754** | **Category 3^†^**  **n=4517** | **Category 4 or 5^‡^**  **n=1503** | **Normal^§^**  **n=5419** |  |
| Predict | Category 1^*^, images (%) | 621 (82) | 193 (4) | 34 (2) | 6 (0) | 854 |
|  | Category 3^†^, images (%) | 110 (15) | 3820 (85) | 269 (18) | 9 (0) | 4208 |
|  | Category 4 or 5^‡^, images (%) | 20 (3) | 483 (11) | 1199 (80) | 2 (0) | 1704 |
|  | Normal^§^, images (%) | 3 (0) | 21 (0) | 1 (0) | 5402 (100) | 5427 |

*, Vienna classification Category 1 includes hyperplastic polyp (HP) or sessile serrated lesion (SSL); †, Category 3 includes low-grade adenoma/dysplasia; ‡, Category 4 or 5 includes high-grade adenoma/dysplasia or submucosal invasive cancer; §, images without lesion.

**Supplementary Table 7:** **Diagnostic performance stratified by modality and manufacturer in the internal validation**

| **WLI**  **Revised Vienna Classification** | **Sensitivity**  **%, (95% CIs)** | **Specificity†**  **%, (95% CIs)** | **Positive predictive value**  **%, (95% CIs)** | **Negative predictive value**  **%, (95% CIs)** | **Accuracy**  **%, (95% CIs)** |
| --- | --- | --- | --- | --- | --- |
| Category 1^*^, images | 80.8  (74.9–86.7) | 99.8  (99.6–100) | 63.8  (57.4-70.1) | 92.8  (91.9-93.8) | 90.7  (89.6-91.7) |
| Category 3^†^, images | 88.9  (87.2–90.7) |  | 90.4  (88.8-92.1) | 90.9  (89.5-92.2) |  |
| Category 4 or 5^‡^, images | 71.1  (66.3–75.8) |  | 76.9  (72.3-81.5) | 92.4  (91.4-93.4) |  |
| Overall, images | 84.6 (82.9–86.3) |  | 84.6  (83.0-86.3) | 99.7  (99.5-100) |  |
| **IEE (NBI/BLI)** | **Sensitivity**  **%, (95% CIs)** | **Specificity†**  **%, (95% CIs)** | **Positive Predictive Value**  **%, (95% CIs)** | **Negative Predictive Value**  **%, (95% CIs)** | **Accuracy**  **%, (95% CIs)** |
| Category 1^*^, images | 86.5  (82.7–90.2) | 99.6  (99.3–99.9) | 74.7  (70.3-79.1) | 92.1  (91.3-92.9) | 90.7  (89.9-91.5) |
| Category 3^†^, images | 85.1  (83.5–86.7) |  | 92.6  (91.4-93.8) | 89.5  (88.4-90.6) |  |
| Category 4 or 5^‡^, images | 82.3  (79.1–85.5) |  | 68.9  (65.4-72.4) | 94.2  (93.5-94.9) |  |
| Overall, images | 84.7 (83.4–86.1) |  | 84.7  (83.4-86.1) | 99.6  (99.4-99.9) |  |
| **Chromoendoscopy** | **Sensitivity**  **%, (95% CIs)** | **Specificity†**  **%, (95% CIs)** | **Positive Predictive Value**  **%, (95% CIs)** | **Negative Predictive Value**  **%, (95% CIs)** | **Accuracy**  **%, (95% CIs)** |
| Category 1^*^, images | 76.7  (71.1–82.3) | 99.8  (99.5–100) | 84.8  (79.9-89.8) | 88.0  (86.8-89.2) | 87.8  (86.6-88.9) |
| Category 3^†^, images | 78.4  (75.9–80.8) |  | 87.7  (85.7-89.8) | 87.8  (86.4-89.2) |  |
| Category 4 or 5^‡^, images | 83.9  (80.8–86.9) |  | 68.2  (64.7-71.7) | 93.3  (92.3-94.3) |  |
| Overall, images | 79.8 (78.0–81.6) |  | 80.2  (78.4-82.1) | 99.0  (98.4-99.5) |  |
| **Manufacturer, Fujifilm** | **Sensitivity**  **%, (95% CIs)** | **Specificity†**  **%, (95% CIs)** | **Positive Predictive Value**  **%, (95% CIs)** | **Negative Predictive Value**  **%, (95% CIs)** | **Accuracy**  **%, (95% CIs)** |
| Category 1^*^, images | 89.0  (82.9–95.1) | 99.5  (98.9–100) | 52.7%  (45.1-60.2) | 94.0  (92.6-95.4) | 88.4  (86.6-90.2) |
| Category 3^†^, images | 83.0  (79.7–86.3) |  | 90.6  (87.9-93.3) | 87.1  (84.7-89.4) |  |
| Category 4 or 5^‡^, images | 68.5  (61.0–76.0) |  | 87.0  (80.8-93.1) | 88.5  (86.6-90.4) |  |
| Overall, images | 81.0  (78.1–83.8) |  | 81.3  (78.5-84.1) | 98.8  (97.8-99.8) |  |
| **Manufacturer, Olympus** | Sensitivity  %, (95% CIs) | Specificity†  %, (95% CIs) | Positive Predictive Value  %, (95% CIs) | Negative Predictive Value  %, (95% CIs) | Accuracy  %, (95% CIs) |
| Category 1^*^, images | 81.3  (78.4–84.3) | 99.7  (99.6-99.9) | 77.8%  (74.7-80.9) | 90.9  (90.3-91.5) | 90.0  (89.4-90.6) |
| Category 3^†^, images | 84.8  (83.6–85.9) |  | 90.8  (89.9-91.8) | 89.5  (88.8-90.3) |  |
| Category 4 or 5^‡^, images | 81.0  (78.9–83.1) |  | 69.2  (66.9-71.4) | 93.9  (93.4-94.4) |  |
| Overall, images | 83.5 (82.6–84.5) |  | 83.6  (82.7-84.6) | 99.5  (99.3-99.7) |  |

*, Vienna classification Category 1 includes hyperplastic polyp (HP) or sessile serrated lesion (SSL); †, Category 3 includes low-grade adenoma/dysplasia; ‡, Category 4 or 5 includes high-grade adenoma/dysplasia or submucosal invasive cancer. CI, confidence interval, Overall sensitivity=AI correctly answered categories 1, 3, and 4 or 5 /GT categories 1, 3, and 4 or 5; Specificity=AI normal /GT normal; Overall PPV=GT categories 1, 3, and 4 or 5/AI answered categories 1, 3, and 4 or 5; Overall NPV= GT normal/AI answered normal; Overall accuracy=AI correctly answered categories 1, 3, and 4 or 5 and normal/All image.

**Supplementary Table 8: Diagnostic performance of the AI system for predicting the pathology of early-stage colorectal cancers and precursor lesions stratified by modality in the external validation**

| Modality | Sensitivity  %, (95% CIs)* | Specificity  %, (95% CIs)* | PPV  %, (95% CIs)* | NPV  %, (95% CIs)* | Accuracy  %, (95% CIs)* |
| --- | --- | --- | --- | --- | --- |
| AI, All  WLI  IEE (NBI/BLI) | 88.3 (82.6–94.1)  88.7 (80.8–96.6)  87.9 (79.5–96.3) | 90.3 (83.0–97.7)  87.9 (76.7–99.0)  93.1 (83.9–100) | 94.6 (90.5–98.8)  93.2 (86.8–99.6)  96.2 (91.1–100) | 80.0 (70.6–89.4)  80.6 (67.6–93.5)  79.4 (65.8–93.0) | 89.0 (84.5–93.6)  88.4 (82.0–94.9)  89.7 (83.3–96.1) |
| Experts, All (n=4)  WLI  IEE (NBI/BLI) | 87.9 (65.0–93.3)  84.7 (62.9–90.3)  91.4 (67.2–96.6) | 72.6 (48.4–90.3)  71.2 (57.6–90.9)  74.1 (37.9–89.7) | 86.5 (77.3–92.9)  85.0 (79.4–92.9)  88.0 (75.3–92.9) | 73.2 (57.1–83.7)  69.9 (56.6–78.6)  78.0 (57.8–90.5) | 79.7 (73.6–84.1)  78.4 (72.6–82.1)  81.0 (74.7–86.2) |
| Fellows, All (n=3)  WLI  IEE (NBI/BLI) | 85.0 (80.8–89.2)  82.3 (75.8–95.2)  86.2 (82.8–87.9) | 66.1 (43.5–71.0)  60.6 (36.4–72.7)  69.0 (51.7–72.4) | 82.9 (75.4–84.3)  79.7 (73.8–83.9)  84.7 (77.4–86.4) | 67.5 (65.7–69.5)  64.5 (61.5–80.0)  71.4 (60.0–75.0) | 77.5 (73.6–78.6)  74.7 (74.7–74.7)  80.5 (72.4–82.8) |
| Novice, All (n=5)  WLI  IEE (NBI/BLI) | 82.5 (75.0–90.0)  77.4 (66.1–88.7)  87.9 (84.5–93.1) | 71.0 (50.0–82.3)  72.7 (63.6–87.9)  72.4 (34.5–79.3) | 84.4 (76.7–90.0)  85.9 (80.0–92.3)  86.0 (74.0–89.5) | 65.8 (59.5–77.8)  60.0 (52.3–77.4)  75.9 (70.0–78.3) | 79.1 (73.1–82.4)  73.7 (67.4–83.2)  81.6 (73.6–85.1) |

CI, confidence interval; PPV, positive predictive value; NPV, negative predictive value; WLI, white-light imaging; NBI, narrow-band imaging; BLI, blue laser imaging: *, Human data shows %, (range).

**Supplementary Table 9: Diagnostic performance of the AI system for predicting the pathology of early-stage colorectal cancers and precursor lesions stratified by endoscope manufacturer in the external validation**

| Manufacturer | Sensitivity  %, (95% CIs) | Specificity  %, (95% CIs) | PPV  %, (95% CIs) | NPV  %, (95% CIs) | Accuracy  %, (95% CIs) |
| --- | --- | --- | --- | --- | --- |
| All | 88.3 (82.6–94.1) | 90.3 (83.0–97.7) | 94.6 (90.5–98.8) | 80.0 (70.6–89.4) | 89.0 (84.5–93.6) |
| Fujifilm | 100 (100–100) | 93.3 (80.7–100) | 96.3 (89.2–100) | 100 (100–100) | 97.6 (92.8–100) |
| Olympus | 85.1 (77.9–92.3) | 89.4 (80.5–98.2) | 94.1 (89.1–99.1) | 75.0 (63.7–86.3) | 86.5 (80.9–92.2) |

CI, confidence interval; PPV, positive predictive value; NPV, negative predictive value; WLI, white-light imaging; NBI, narrow-band imaging; BLI, blue laser imaging.

**Supplementary Table 10: EfficientNet-B8 architecture**

| **Stage** | **Operator** | **Resolution** | **Out Channels** | **Layers** |
| --- | --- | --- | --- | --- |
| 1 | Conv3×3 | 256×256 | 72 | 1 |
| 2 | MBConv1, k3×3 | 128×128 | 32 | 4 |
| 3 | MBConv6, k3×3 | 128×128 | 56 | 8 |
| 4 | MBConv6, k5×5 | 64×64 | 88 | 11 |
| 5 | MBConv6, k3×3 | 32×32 | 176 | 12 |
| 6 | MBConv6, k5×5 | 16×16 | 248 | 14 |
| 7 | MBConv6, k5×5 | 16×16 | 424 | 8 |
| 8 | MBConv6, k3×3 | 8×8 | 704 | 4 |
| 9 | Conv 1×1 & Pooling & FC | 8×8 | 2816 | 1 |
