# Supplementary figures and images for: Robust automated prediction of the revised Vienna Classification in colonoscopy using deep learning: development and initial external validation
Source: J Gastroenterol. 2022 Aug 16;57(11):879–89. doi: 10.1007/s00535-022-01908-1 (PMC9596523; doi:10.1007/s00535-022-01908-1)

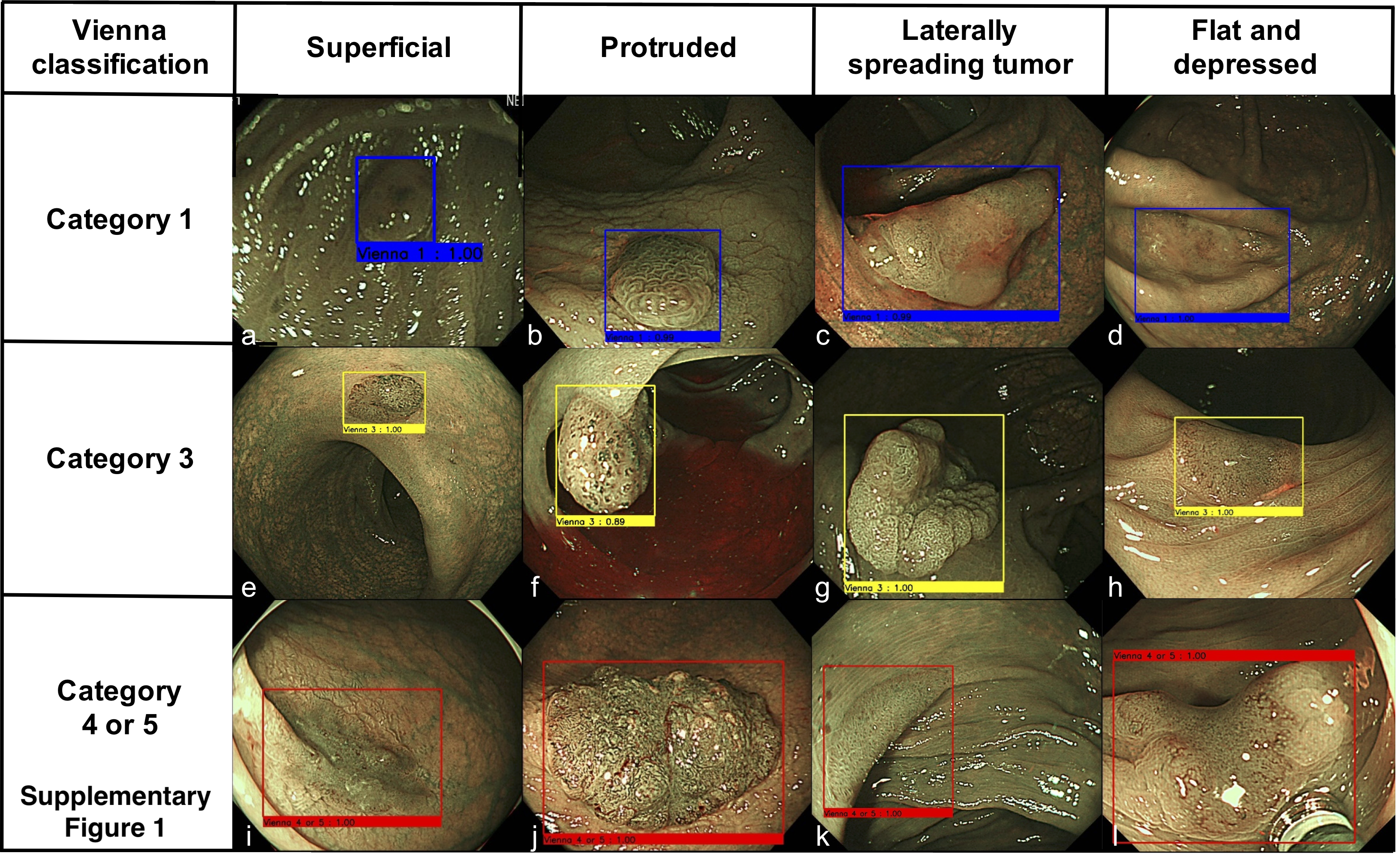

Supplement: Supplementary file 2 — Supplementary file2 (TIFF 9950 KB) [file 535_2022_1908_MOESM2_ESM.tiff]

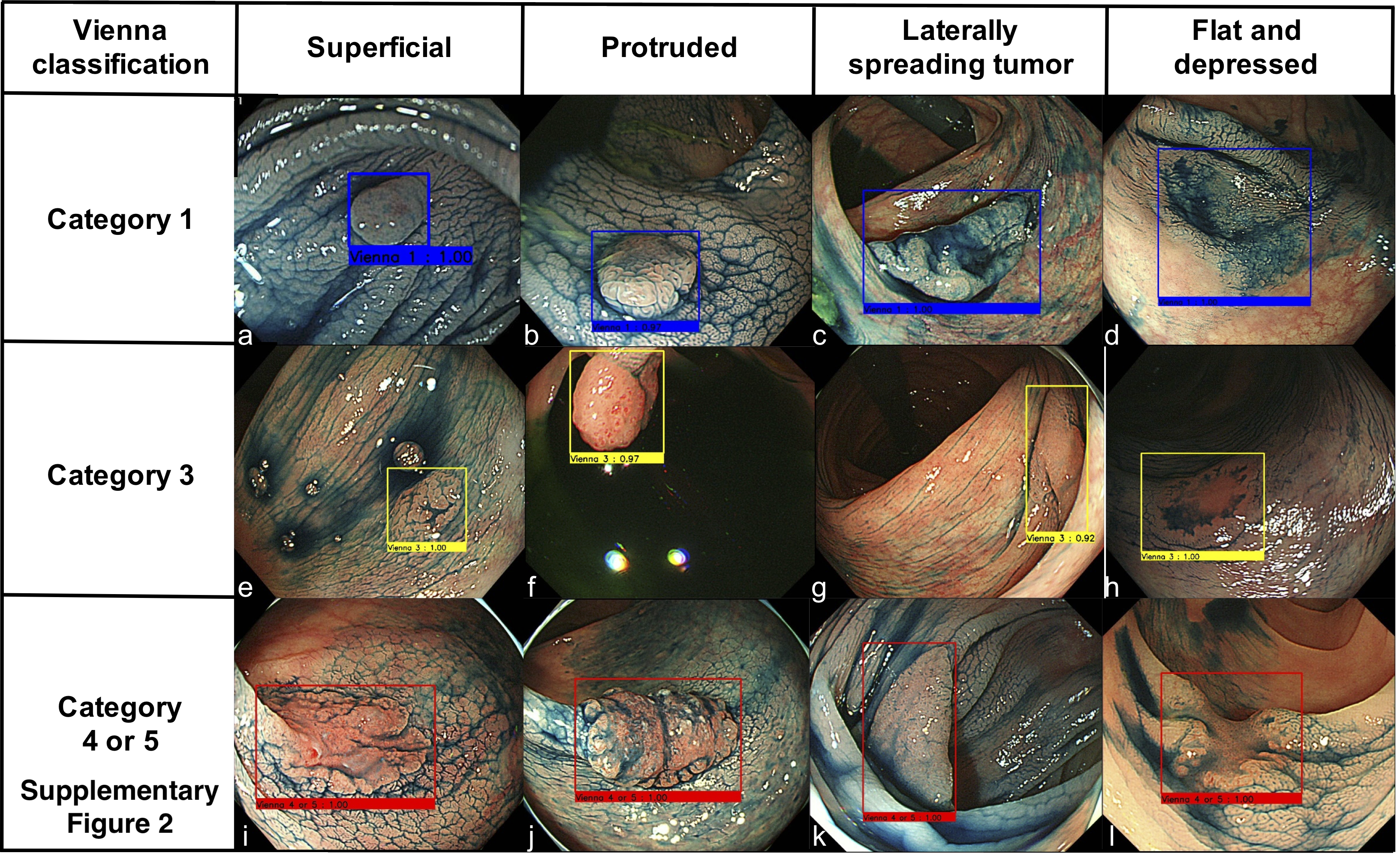

Supplement: Supplementary file 3 — Supplementary file3 (TIFF 25774 KB) [file 535_2022_1908_MOESM3_ESM.tiff]

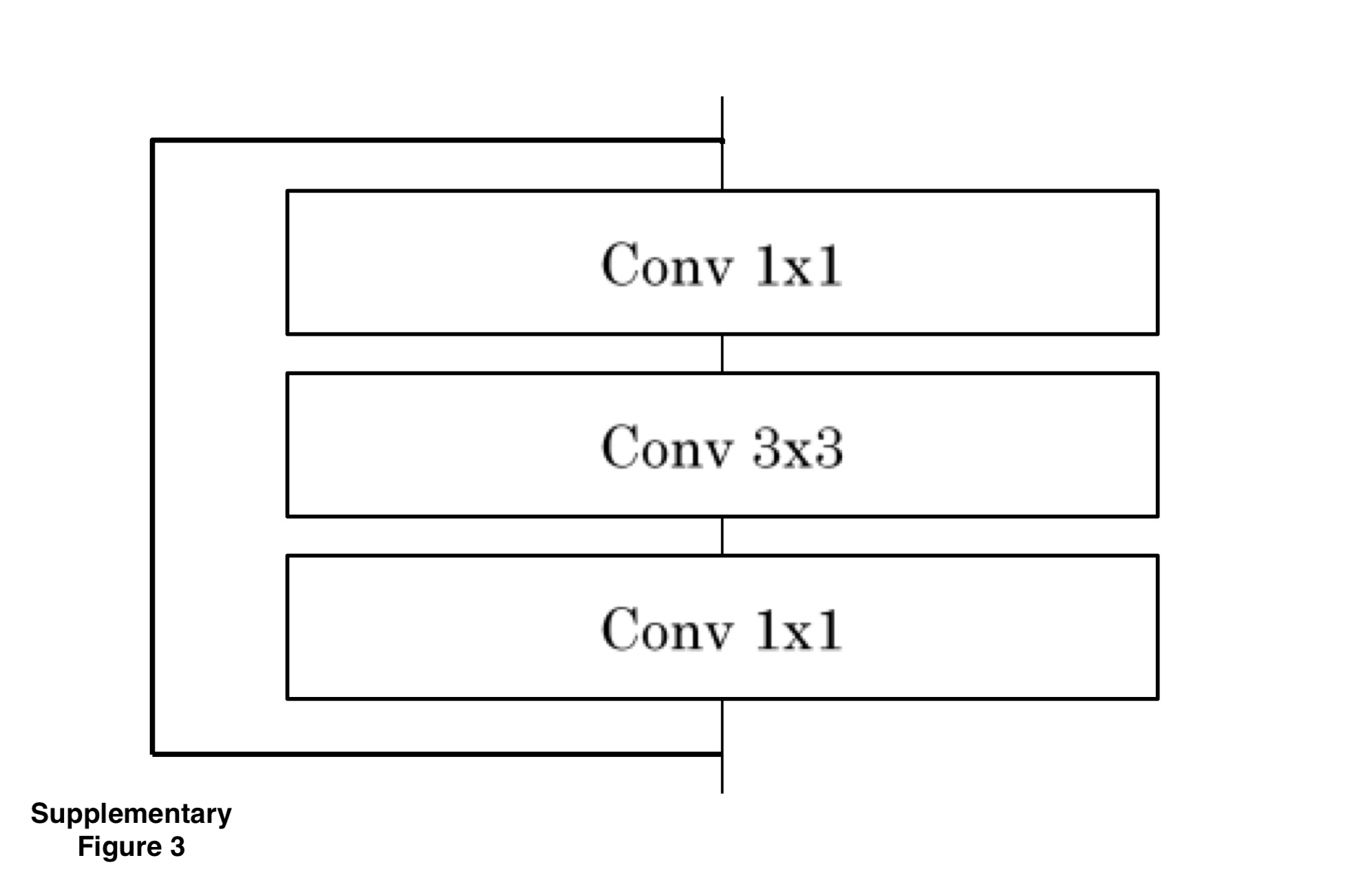

Supplement: Supplementary file 4 — Supplementary file4 (TIFF 184 KB) [file 535_2022_1908_MOESM4_ESM.tiff]

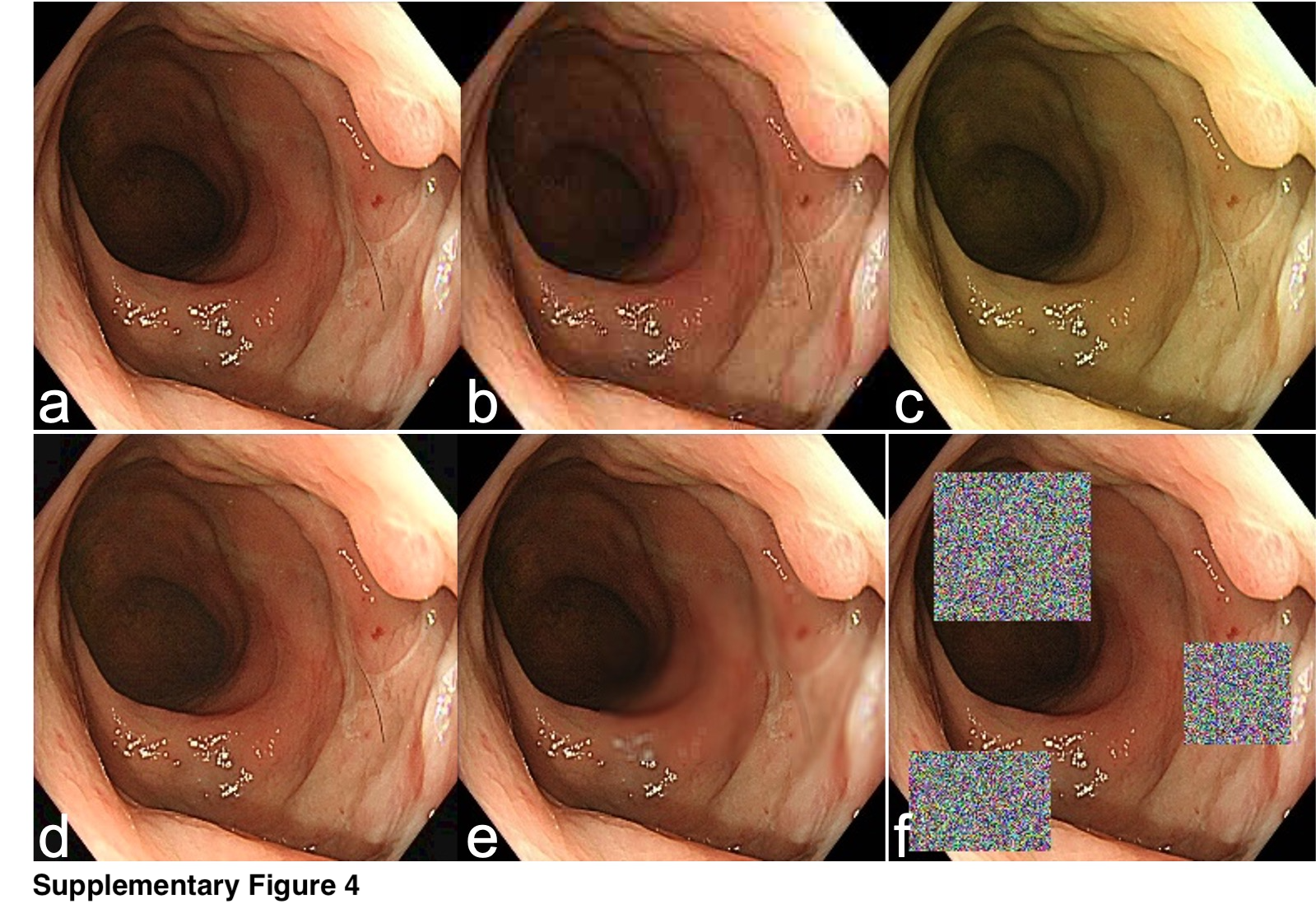

Supplement: Supplementary file 5 — Supplementary file5 (TIFF 2971 KB) [file 535_2022_1908_MOESM5_ESM.tiff]

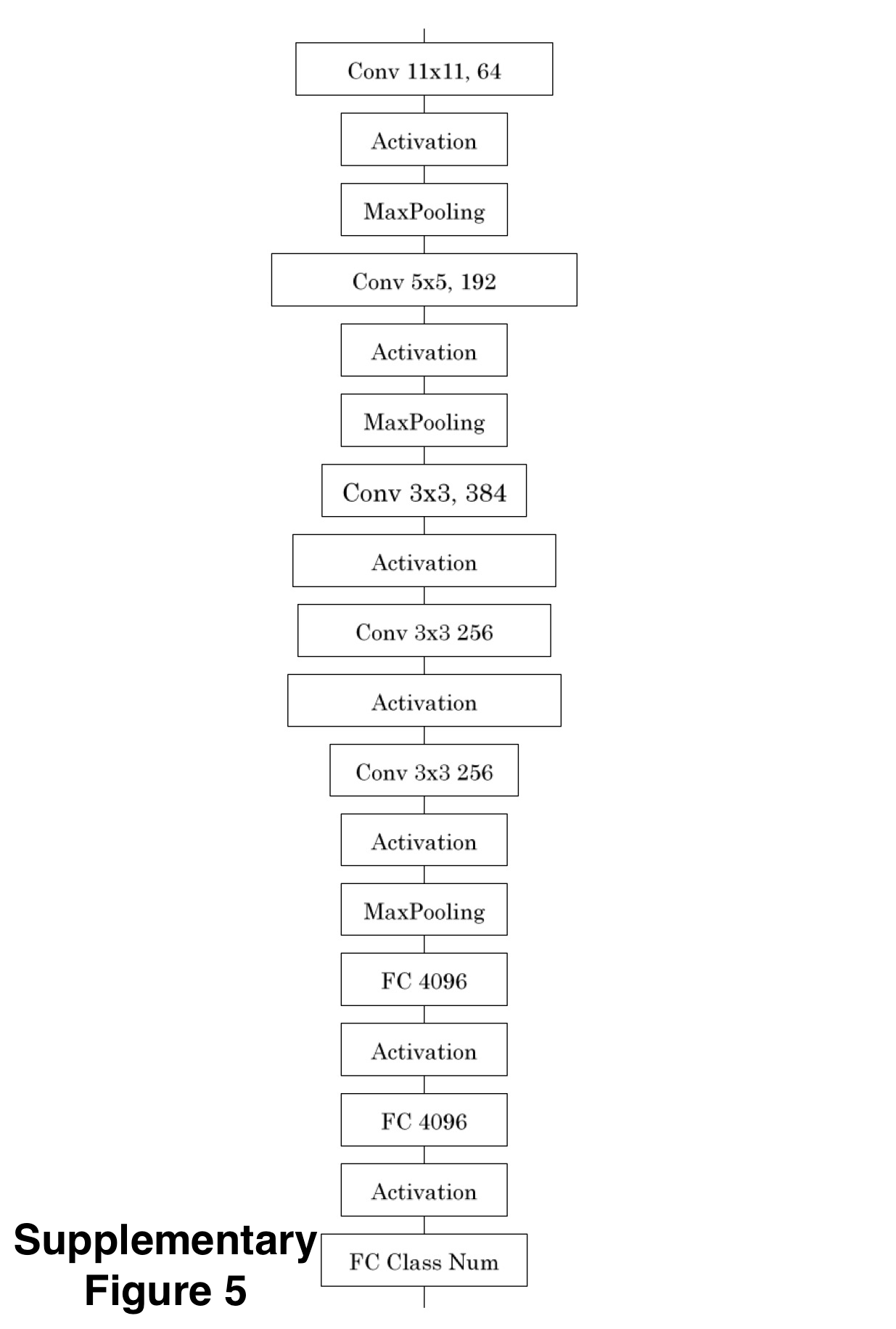

Supplement: Supplementary file 6 — Supplementary file6 (TIFF 250 KB) [file 535_2022_1908_MOESM6_ESM.tiff]

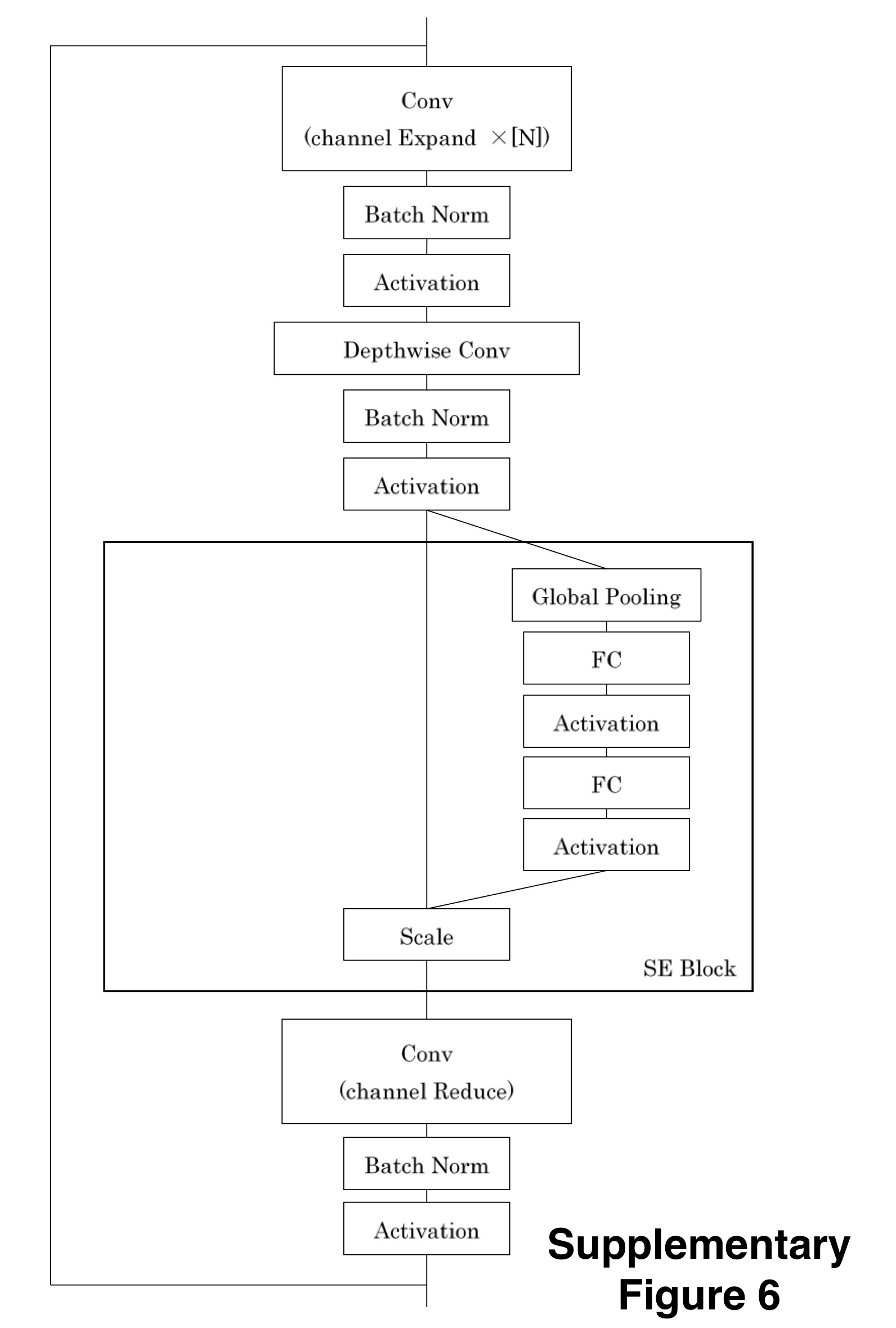

Supplement: Supplementary file 7 — Supplementary file7 (TIFF 394 KB) [file 535_2022_1908_MOESM7_ESM.tiff]

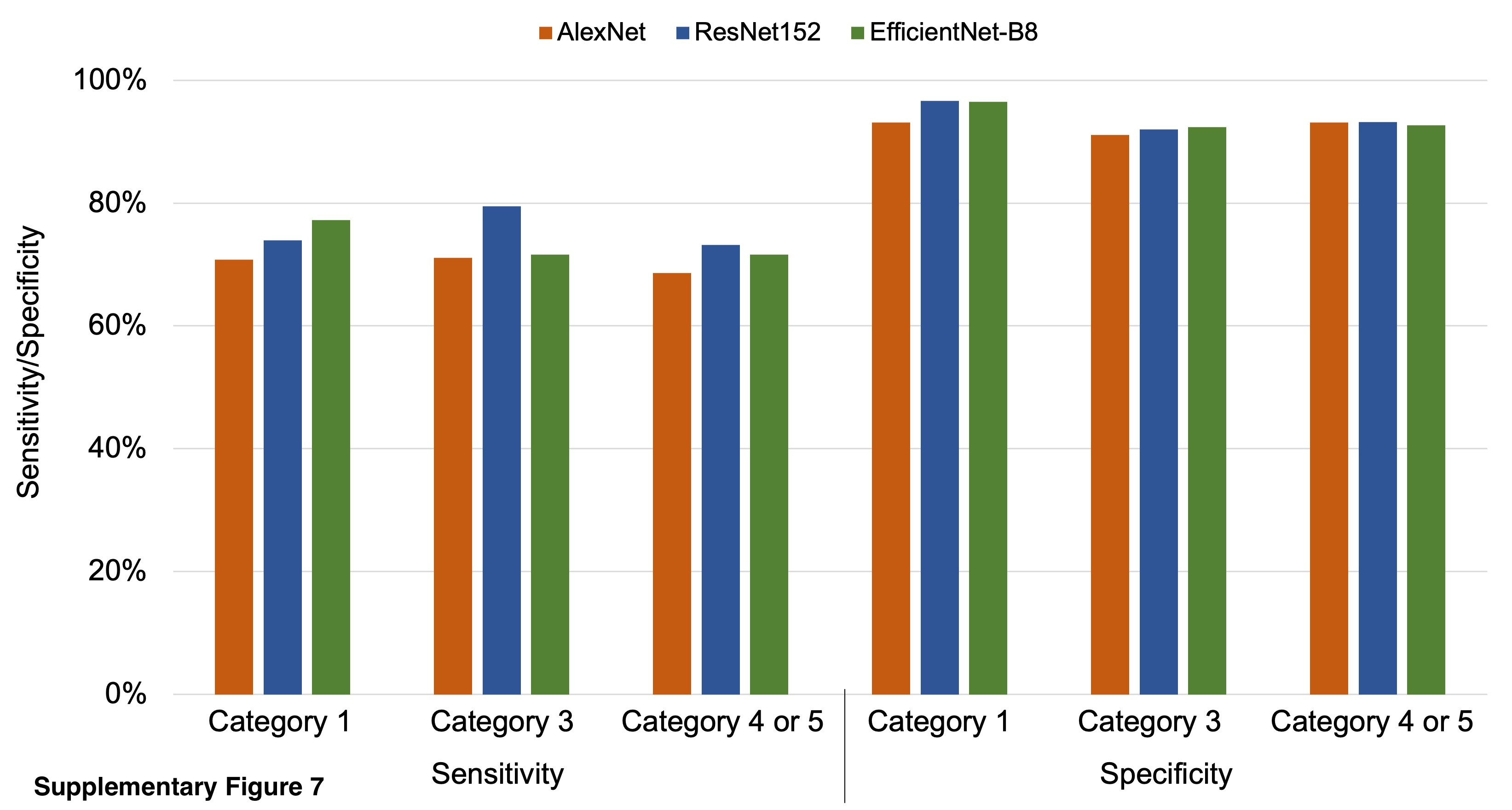

Supplement: Supplementary file 8 — Supplementary file8 (TIFF 1295 KB) [file 535_2022_1908_MOESM8_ESM.tiff]
